# Histone-like nucleoid structuring (H-NS) protein silences the beta-glucoside (*bgl*) utilization operon in *Escherichia coli* by forming a DNA loop

**DOI:** 10.1016/j.csbj.2022.11.027

**Published:** 2022-11-12

**Authors:** Katie Jing Kay Lam, Zhongge Zhang, Milton H. Saier

**Affiliations:** Department of Molecular Biology, University of California at San Diego, La Jolla, CA, USA

**Keywords:** Transcriptional regulation, Beta-glucoside (*bgl*) operon, Silencing expression, Repression by H-NS, DNA looping

## Abstract

The *bgl* operon of *Escherichia coli* encodes proteins mediating the metabolism of aromatic beta-glucosides, but the operon is silent in wild type cells. Insertion of an insertion sequence (IS) element in the regulatory region upstream of the *bgl* promoter activates expression of the operon. The repression mechanism involves the histone-like nucleoid structuring (H-NS) protein with two DNA binding sites, one in the region upstream of the promoter, and the other within the first structural gene of the operon, *bglG*. The detailed mechanism of repression is not well understood. Here, we first show two terminators flanking *bglG* are not required for *bgl* operon silencing. Instead, several lines of experimental evidence clearly suggest that the silencing mechanism involves looping of the DNA between H-NS’s two DNA binding sites. H-NS is known to preferentially bind to AT-rich curved DNA, and such regions are found in the vicinity of both sites. We show that strong repression is abolished by (1) preventing H-NS self-oligomerization while retaining DNA binding, (2) preventing or reducing H-NS binding to the *bgl* operon regulatory region, and (3) preventing or reducing H-NS binding to the binding site in the *bglG* gene. We also show that the phase of the DNA between these two binding sites is not important, and that large insertions of DNA in the putative loop region do not diminish repression. These results imply that H-NS depends on DNA looping to exert strong repression.

## Graphical abstract

**Figure:**
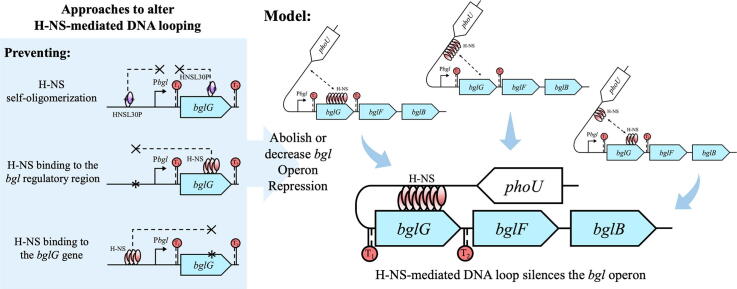

## 1 Introduction

The β-glucoside (*bgl*) utilization operon in *Escherichia coli* (*E. coli*) encodes necessary proteins for the uptake and utilization of aromatic β-glucosides such as salicin, esculin and arbutin [1], [2]. The operon is silent in wild-type *E. coli* strains such that cells are not able to grow on these β-glucosides as sole carbon sources. In spite of this, the *bgl* operon can be activated by mutations, and it then becomes inducible in the presence of β-glucosides, so that *E. coli* cells can utilize β-glucosides for growth, leading to the Bgl positive (Bgl^+^) phenotype. One of the most common types of mutations giving rise to the Bgl^+^ phenotype is the transposition of IS elements like IS1 and IS5 into the *bgl* regulatory region [2], [3]. In addition, the operon can be activated by non-insertional mutations in the *bgl* regulatory region or outside the *bgl* operon, which counteract the silencing of the operon [4], [5], [6], [7], [8], [9], [10], [11].

The *bgl* operon includes three genes, *bglG*, *bglF* and *bglB*, with two rho-independent terminators flanking *bglG* [2], [12]. In the absence of β-glucosides, transcription is terminated at the two terminators due to the formation of hairpin-like secondary structures. However, in the presence of β-glucosides, the product of the first gene, *bglG,* acts as a transcriptional antiterminator which binds to an upstream region that partially overlaps each terminator. This prevents the formation of the hairpin-like structures of the two terminators, thus relieving the termination and allowing full length transcription of the operon [2], [13], [14], [15]. The second gene, *bglF*, encodes an enzyme II (EII) of the phosphoenolpyruvate:sugar phosphotransferase system (PTS), which is responsible for the transport and phosphorylation of extracellular β-glucosides [16], [17], [18]. The BglF protein can also phosphorylate and dephosphorylate BglG, regulating BglG antitermination [12], [18]. In the presence of β-glucosides, BglG is dephosphorylated by EII and phosphorylated at a different site by a phosphocarrier protein (HPr) of the PTS, which leads to full activity of BglG-mediated antitermination [12], [18], [19], [20], [21]. The third gene, *bglB*, encodes a phospho-β-glucosidase which hydrolyzes the aglycon-sugar linkage in the phospho-β-glucosides that are transported and phosphorylated by BglF [2], [22]. Even though the two terminators terminate transcription, and the activated BglG prevents termination, it is still unclear if the terminators contribute to and play a significant role in the silencing of the *bgl* operon.

There are several transcriptional regulators reported to be involved in upregulating expression of the *bgl* operon, such as the cyclic-AMP receptor protein (Crp), LeuO and BglJ [6], [7], [8], [10], [11]. Crp is a global regulator, which once bound to cyclic-AMP (cAMP), activates the *bgl* operon promoter by binding to a site in the regulatory region [23], [24]. A single mutation in the Crp operator, creating a stronger Crp-cAMP binding site, leads to transcriptional activation of the operon [6]. In addition, LeuO and BglJ are transcriptional regulators which when present at high (overexpressed) levels, also bind to the *bgl* regulatory region and increase the expression of the *bgl* operon [7], [8], [10]. BglJ forms heterodimers with another transcriptional regulatory protein RcsB, which binds to the *bgl* regulatory region and activates the operon [11]. Both LeuO and BglJ, when overexpressed, relieve repression of the *bgl* operon when bound to the *bgl* regulatory region, but these transcription factors have no effect on the region downstream of the promoter [10].

As mentioned above, the *bgl* operon is silent in wild-type *E. coli* cells. The major repressor of the *bgl* operon is the histone-like nucleoid structuring protein (H-NS), which is involved in chromosomal organization and acts as a global negative regulator [4], [9], [23], [25], [26]. H-NS preferentially binds to AT-rich and curved DNA regions [27]. It consists of a dimerization/oligomerization domain at the N-terminus and a DNA-binding domain at the C-terminus [28], [29]. Once bound to DNA, H-NS can form higher order oligomers [30]. H-NS-mediated nucleoprotein complexes are believed to be important for effective gene repression and silencing. This protein acts by bridging DNA duplexes through H-NS oligomerization, thus allowing the formation of DNA loops [31], [32]. The formation of such nucleoprotein complexes prevents transcription by either trapping RNA polymerase or blocking RNA polymerase from binding to the promoter region [33]. However, the change of leucine to proline at the 30th codon (L30P) position within the N-terminal domain of the protein abolishes H-NS’s ability to form higher order oligomers while still retaining its ability to form dimers and bind to DNA [34].

In relation to the *bgl* operon, H-NS binds close to the Crp-cAMP binding site within the regulatory region, as well as a downstream region within the *bglG* gene [6], [9], [35]. However, the exact H-NS binding sites in the *bgl* operon are still not defined. In addition, it is still unclear what the exact mechanism is by which H-NS silences the *bgl* operon, although it is known that the binding of H-NS to both locations is required for full repression, and the repression involving H-NS at these two sites is synergistic [36]. A missense H-NS mutant carrying an amino acid substitution at position 30 (Leu30 to Pro or L30P), when expressed in a plasmid, is thought to be deficient in dimerization, thereby losing the ability to function as a transcriptional repressor [29]. In another report, this same H-NS mutant was reported to maintain its dimerization and DNA-binding property [34]. Also, when H-NS is truncated at the N-terminus with loss of its DNA binding ability, another repressor of the *bgl* operon, StpA can function as a DNA-binding adapter of H-NS to repress the operon [37], [38]. However, StpA is negatively regulated by H-NS and can only regulate the *bgl* operon when H-NS is absent [39], [40]. Therefore H-NS is the major repressor of the *bgl* operon in wild-type *E. coli* cells.

DNA looping, mediated by transcription factors, is a mechanism sometimes used for strong and effective regulation of gene transcription [41]. For the *gal* operon, the binding of the galactose repressor (GalR) to the two operators in the *gal* operon alone is insufficient to repress the operon. Instead, the formation of a DNA loop, resulting from the protein–protein interaction between GalR proteins, is required for strong repression of the *gal* operon [42]. Also, a similar DNA looping mechanism has been shown in the *E. coli lac* operon, with the binding of the *lac* repressor, LacI, to two of the three *lac* operators within the operon [43]. The tetrameric repressor CI of Lambda phage binds to its two operators O_R_ and O_L_ (separated by 2.3 kb) and loops them together by octamerization when the phage is incorporated into the *E. coli* chromosome [44], [45]. The length of the intervening DNA in the DNA loop can be as long as 5 kb [46]. Please note that all these repressors have their specific operators (DNA binding sites). Each DNA loop consists of the intervening DNA between two operators and a fixed oligomer (a dimer for GalR, a tetramer for LacI, and an octamer for CI) [42], [43], [44], [45].

As a common repressor, H-NS can exert its repressive effect on transcription by first binding to two or more DNA targets and then looping these target sites, trapping RNA polymerase at the promoter or excluding binding of RNA polymerase from its operator [33], [47]. Such a repression loop has been visualized and reported in previous studies on the transcriptional regulation of the *hdeAB* and *rrnB* operons in *E. coli* [33], [48]. Once an H-NS dimer binds to a target site, it undergoes self-oligomerization, forming a stiffened filament along the DNA strand [49]. The formation of such an H-NS/DNA nucleoprotein complex (that is, a stiffened filament) is required for efficient repression [50]. Likely, a similar mechanism applies to the repression of the *bgl* operon by H-NS, but this has not been demonstrated directly. Our prior studies have suggested that H-NS binding to its binding site(s) in the *bgl* promoter region is not sufficient to repress the operon, so we proposed that H-NS represses the *bgl* operon by the formation of a DNA loop between its two binding sites [39]. This proposed mechanism of *bgl* operon repression was investigated in-depth in this study.

Here, we first show both terminators flanking the *bglG* gene are not required for *bgl* operon silencing as the operon remains uninducible when these terminator sequences are deleted. We then examine the effect of preventing the formation of the H-NS-mediated DNA loop on *bgl* operon transcriptional activity, as well as the ability of *E. coli* cells to grow on aromatic β-glucosides. In order to do so, the formation of the DNA loop was altered using the following approaches: (1) preventing H-NS self-oligomerization, (2) preventing or reducing H-NS binding in the *bgl* operon regulatory region, (3) preventing or reducing H-NS binding within the *bglG* gene, (4) changing the phase of the DNA between the two H-NS binding sites, and (5) changing the distance between the two H-NS binding sites. We demonstrate that H-NS binding to both upstream and downstream sites simultaneously is insufficient to abolish transcription. However, a DNA loop between these binding sites, formed by H-NS oligomerization, is conceivably required for full repression. The proposed H-NS-mediated DNA loop is robust as it is independent of DNA phasing and the length of intervening DNAs. The presence of such a strong DNA loop would explain why other transcription factors (reported to be involved in *bgl* regulation), when expressed at wild type levels, have negligible effects on *bgl* operon expression. These results provide a new perspective on the mechanism by which the *bgl* operon is repressed by H-NS in wild-type *E. coli* cells.

## 2 Results

### 2.1 Rho-independent terminators are not required for *bgl* operon silencing

**Fig. 1:**
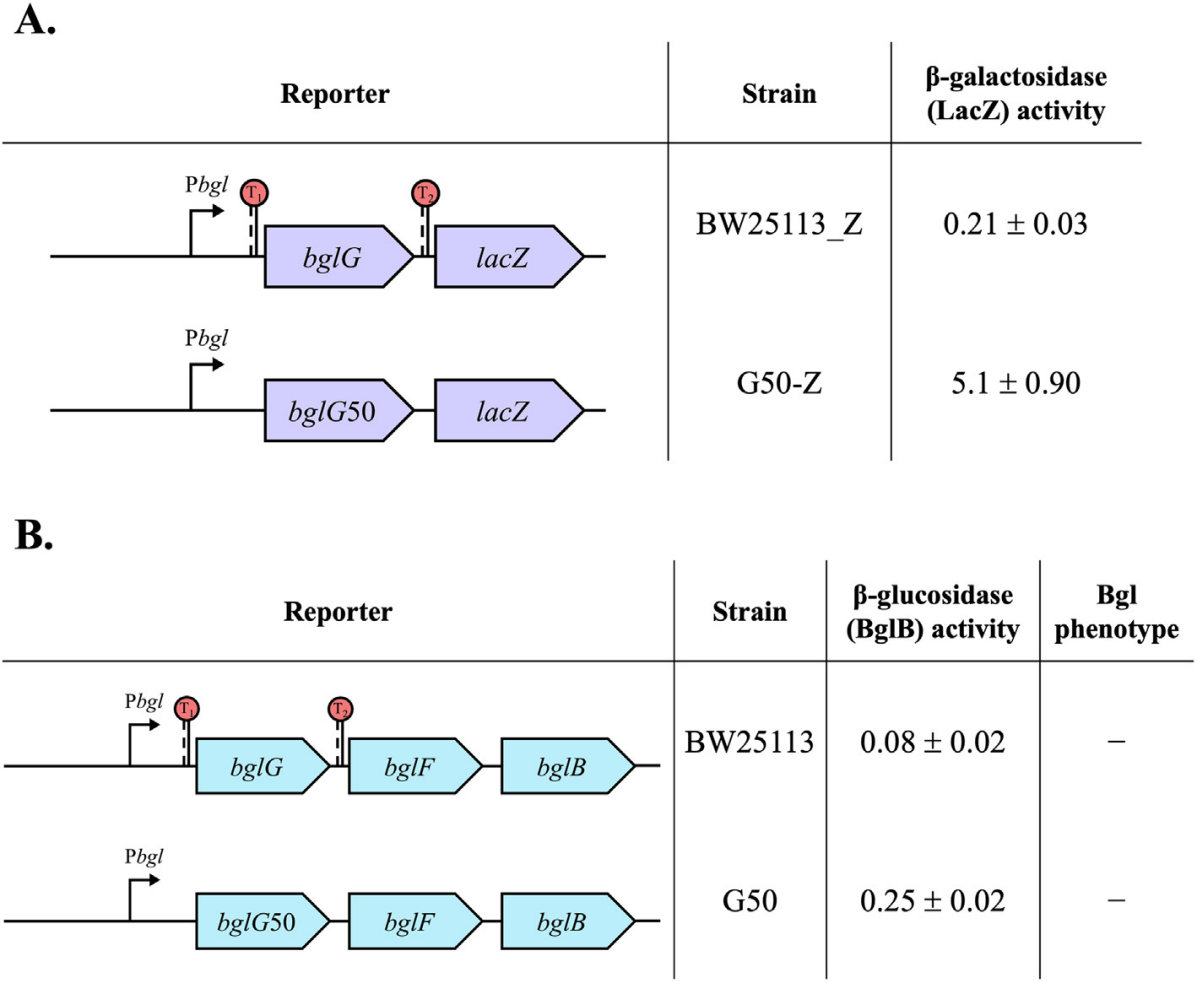
The Rho-independent terminators flanking the *bglG* gene are dispensable for *bgl* operon silencing. (A) Effects of deleting both terminators on *bgl* operon expression using β-galactosidase (LacZ) reporter assays. The left panel represents two transcriptional *lacZ* reporters used to examine expression of the *bgl* operon with (BW25113_Z) or without (G50-Z) terminators (T_1_ and T_2_) flanking *bglG* at the *lac* locus. The right panel shows the β-galactosidase (LacZ) activities representing the *bgl* operon activities. Bacterial samples were subject to β-galactosidase (LacZ) assays as described in Materials and Methods. (B) Effects of deleting both terminators on *bgl* operon expression using the β-glucosidase (BglB) assay. The left panel represents the *bgl* operon with (BW25113) or without terminators (G50). The right panel shows the β-glucosidase (BglB) activities of both wild type and strain G50 and their Bgl phenotypes.

The first gene of the *bgl* operon, *bglG*, is flanked by two Rho-independent terminator sequences. To determine if these terminators play a role in silencing the *bgl* operon, we first removed both terminators from our *bgl* operon reporter, P*bgl-bglG*-*lacZ* strain [51], creating a new operon reporter strain, G50-Z. As shown in Fig. 1A, the new operon reporter (without terminators) showed a slight increase in β-galactosidase (LacZ) activity compared to BW25113_Z.

The two terminators were also removed from the native *bgl* operon, yielding strain G50. As seen in Fig. 1B, the β-glucosidase (BglB) activity of strain G50 (with no terminators) was similar to that of the wild-type, although with a slight increase. The G50 strain also showed a Bgl^−^ phenotype, as its colonies did not show a visible red color after 1 day of incubation on MacConkey indicator plates with 0.5% salicin at 37°C, indicating its inability to ferment salicin. Also, no visible colonies were seen on M9 minimal agar plates with salicin after 2 days of incubation at 37°C, which further confirmed its Bgl^−^ phenotype. All of these results showed that the *bgl* operon remains silent (and non-inducible by salicin) in the absence of both terminators flanking the *bglG* gene. This terminator-less strain was used in most of the experiments in this study.

### 2.2 H-NS oligomerization is necessary for *bgl* operon silencing

In an earlier paper, we proposed that H-NS represses the *bgl* operon by forming a DNA loop between its two binding sites [39]. Here, we investigated this proposed DNA-looping mechanism in-depth. To form a DNA loop, H-NS proteins have to bind to the upstream site and the downstream site simultaneously and then perform self-oligomerization to bridge the two sites together. We examined the effects of altering each of these three factors (upstream binding, downstream binding and self-oligomerization) on formation of such a loop by measuring *bgl* operon activity.

**Fig. 2:**
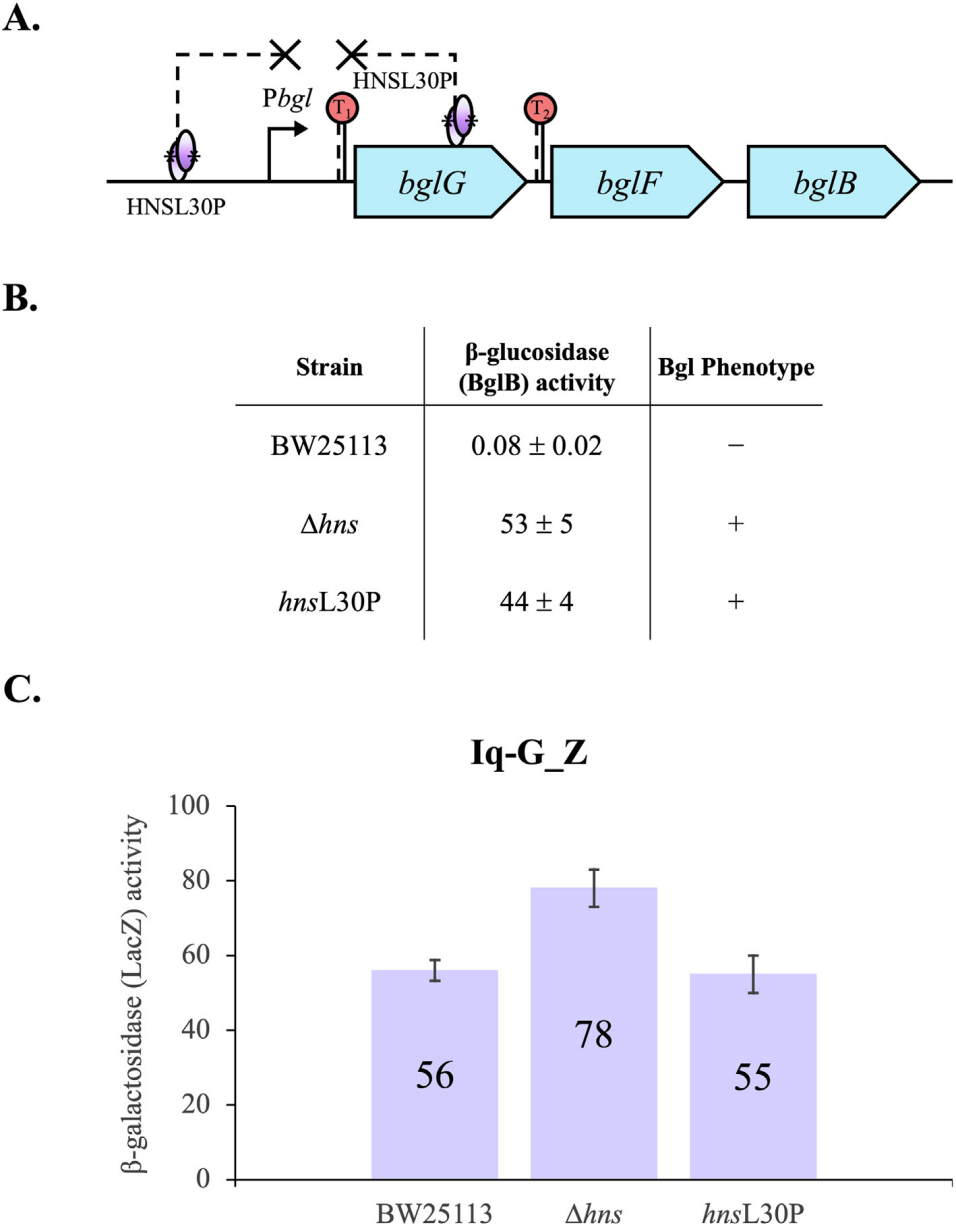
H-NS self-oligomerization is essential for *bgl* operon silencing. (A) Diagram showing the binding of H-NSL30P to upstream and downstream sites within the native *bgl* operon. As an H-NS derivative, H-NSL30P carries a proline residue instead of leucine at the 30th residue position and still maintains its DNA binding capability while being deficient in oligomerization [34], thereby failing to bridge two or more DNA loci. (B) *bgl* operon activities and Bgl phenotypes of strains BW25113 (wild type H-NS), Δ*hns* and *hns*L30P. (C) Repressive effects of H-NS binding and H-NSL30P binding on *bglG* expression. The cassette, a *lacIq* constitutive promoter (Iq) driving the *bglG* gene (Iq-G), was fused upstream of *lacZ*’s RBS at the *lac* locus. The resultant reporter was used to determine the inhibitory effects of H-NS and H-NSL30P on *bglG* expression by measuring β-galactosidase (LacZ) activities. Lower β-galactosidase activities should be seen if H-NS or H-NSL30P binds within the *bglG* gene.

We first determined the effect by preventing the oligomerization of H-NS using the *hns*L30P mutant (a missense mutation in the *hns* gene leading to a change of leucine to proline at the 30th codon) (Fig. 2A) [29], [39]. This derivative of H-NS is still capable of dimerizing, thereby binding to the target DNA, but it has lost the ability to oligomerize [34]. Therefore, possibly, this modified H-NS is unable to form nucleoprotein complexes involving DNA bridges and DNA looping. Fig. 2B reveals that the operon activity (44 units) of *hns*L30P increased 550 times compared to strain BW25113 (0.08 units) which expresses the wild type H-NS, indicating this H-NSL30P mutant protein lost most of the repressive effect on the operon. However, the operon activity of *hns*L30P was lower (by 53 units) than that of the Δ*hns* mutant (Fig. 2B). Most likely this is attributable to the binding of H-NSL30P to both the P*bgl* and *bglG* binding sites for H-NS, leading to some repression (see below). Both Δ*hns* and *hns*L30P showed Bgl^+^ phenotypes on both MacConkey + salicin plates and M9 + salicin plates.

Our previous results have shown that H-NS and H-NSL30P are able to bind to the *bgl* promoter region in the absence of *bglG*, while H-NSL30P even showed a higher binding affinity [39]. Here, we further demonstrated H-NS binding to its downstream site within *bglG*, using the *lacIQ* promoter driving expression of the *bglG*-*lacZ* transcriptional fusion at the *lac* locus (Iq-G_Z). As seen in Fig. 2C, using the Iq-G_Z reporter, the presence of H-NS (strain BW25113) or H-NSL30P (strain *hns*L30P) led to lower LacZ activity than the absence (Δ*hns*) of either protein. On the other hand, both H-NS and H-NSL30P have similar repressive effects on *bglG* expression. These results confirmed that H-NSL30P retains the ability to bind to *bglG* like the wild-type H-NS, despite its inability to oligomerize. Together with our previous observations [39], this H-NS derivative, H-NSL30P, is still able to bind to its two sites although it has lost its oligomerization property. These data also indicate that H-NS binding to *bglG* alone (without DNA looping) only partially represses *bgl* operon expression.

A stronger promoter, P*tet,* driving *bglG*-*lacZ* expression (P*tet*-G-Z) was also tested for possible inhibitory effects of H-NS and H-NSL30P. However, no obvious operon repression was observed when H-NS or H-NSL30P was present (data not shown). This might be due to the fact that H-NS repression, due to binding to the *bglG* gene, is less efficient when a stronger promoter is used [36]. With a strong promoter like P*tet*, the RNA polymerase might be able to transcribe through the H-NS binding site when H-NS or H-NSL30P is bound at this site. This explains our results, suggesting that H-NS repression is seen in the background of wild-type *hns* and mutant *hns*L30P when a moderate promoter (Iq-G_Z) is used, but not when a stronger promoter (P*tet*-G-Z) is used.

### 2.3 H-NS binding to the *bgl* operon regulatory region is necessary for strong *bgl* repression

**Fig. 3:**
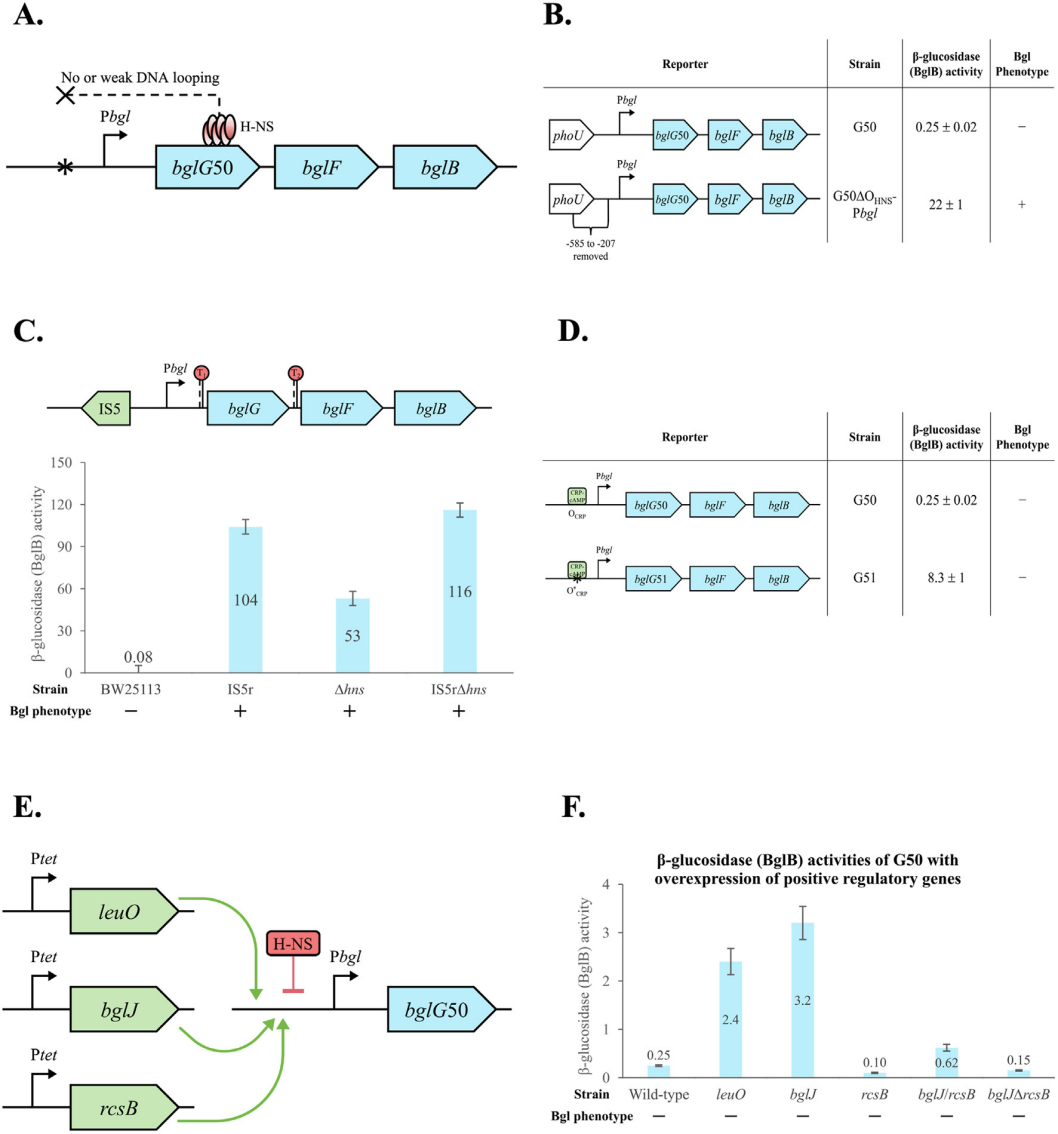
Preventing formation of the DNA loop by abolishing or decreasing H-NS binding to its upstream site within the *bgl* regulatory region. Strain G50 with deletion of both terminators flanking *bglG* was used in most of the experiments, but a Bgl^+^ mutant (IS5r) in the wild type background was used in one experiment. (A) Diagram showing no or weak H-NS binding to the upstream site. Four approaches used to impede H-NS binding included deletion of the upstream region, IS5 insertion, stronger Crp binding, and overexpression of *bgl* positive regulators. (B) Preventing H-NS binding to its upstream site by removing an upstream region carrying the proposed binding site. The left panel shows two operon reporters. The right panel shows the β-glucosidase (BglB) activities and the Bgl phenotypes. (C) Preventing H-NS binding to its upstream site by IS5 insertion. An IS5 insertion-mediated Bgl^+^ mutant was used in these experiments. The upper panel shows insertion of an IS5 element upstream of P*bgl*, abolishing H-NS binding. The lower panel shows the operon (BglB) activities and the Bgl phenotypes. (D) Weakening H-NS binding to its upstream site by increasing Crp binding. Strain G51, harboring a “C” to “T” substitution at position −67 (relative to the *bglG* transcriptional start site) within the Crp operator (thus yielding a stronger Crp binding site, O_CRP_*), was used in these experiments. The left panel shows two operon reporters with the wild type (O_CRP_) and the stronger (O_CRP_*) Crp binding sites, respectively. The right panel shows the operon (BglB) activities and the Bgl phenotypes. (E) Diagram showing reduced H-NS binding to the upstream site by overexpression of *leuO*, *bglJ* and *rcsB* on the chromosome. (F) Effects of overexpressing *leuO*, *bglJ* and *rcsB* on *bgl* operon activities. A strong constitutive promoter, P*tet*, was individually substituted for the native promoter of each target gene on the chromosome, leading to high levels of expression. For strain P*tet*-*bglJ*/*rcsB*, both genes are driven by P*tet* simultaneously.

Next, we sought to prevent H-NS-mediated DNA looping by decreasing or abolishing H-NS binding to the upstream site of the *bgl* operon regulatory region (Fig. 3A). The approaches included: (1) deletion of an upstream region carrying the proposed H-NS binding site; (2) insertion of IS elements upstream of P*bgl* where the upstream H-NS binding site is thought to be, and (3) overexpression of *bgl* operon positive regulators.

Fig. 3B shows that strain G50ΔO_HNS_-P*bgl* (deleted for the upstream region, where the H-NS binding site is thought to be, in front of the Crp operator) exhibited a 90-fold greater operon activity than strain G50, in which H-NS can bind to both the upstream and downstream sites. This fact clearly indicates that with no binding (or a significantly decreased binding) to the upstream site, H-NS almost completely loses its repressive effect on *bgl* operon expression. As expected, strain G50ΔO_HNS_-P*bgl* is Bgl^+^.

Fig. 3C shows the *bgl* operon activity of an IS5 insertional mutant, IS5r, in which an IS5 element (in the reverse orientation) was inserted upstream of the Crp operator in the *bgl* regulatory region of BW25113. Compared to the wild-type strain, IS5 insertion dramatically elevated the operon activity (see columns 1 and 2). Furthermore, the IS5 insertional mutant, IS5r, shows an almost 2-fold increase in operon activity compared to the *hns* deletion mutant (Δ*hns*). This may be because without H-NS, the other repressor, StpA is expressed at a greater level and therefore exerts a repressive effect on *bgl* operon expression [39]. This was confirmed using our β-glucosidase (BglB) assay. As shown in Figure S1, the *stpA* mutation had no effect on *bgl* operon activity, probably due to the strong H-NS repression of *stpA* expression. However, *bgl* operon transcription of the *hns*/*stpA* double mutant (Δ*hns*Δ*stpA*) increased 2-fold compared to the *hns* single mutant, confirming that StpA exhibits an inhibitory effect on *bgl* operon expression in the absence of H-NS. On the other hand, the *hns* and IS5 insertional double mutant (IS5rΔ*hns*) showed a slightly higher operon activity than that of the IS5 insertional mutant alone (see columns 2 and 4 in Fig. 3C). It is possible that IS insertions not only prevent H-NS binding to the *bgl* regulatory region, but also generate new promoters, or attract new regulators to the *bgl* operon. As expected, the IS5r mutant showed a Bgl^+^ phenotype on both MacConkey + salicin plates and M9 + salicin plates.

We further examined the effects of IS varieties, locations and orientations on alteration of H-NS binding to the *bgl* regulatory region. Fig. S2A shows that IS1 elements tended to be inserted further from the *bglG* transcriptional start site, followed by IS5 elements, with IS2 elements being closest to the *bglG* transcriptional start site. However, more data are needed to support this preliminary observation. Also, the same IS elements inserted in different orientations (direct or reverse) at the same location showed different operon activities (Fig. S2B). These results indicate that although insertions of IS1, IS2 and IS5 all activated the *bgl* operon, leading to a Bgl^+^ phenotype, the activation levels by IS insertions are not closely correlated with the type of IS elements, the insertional positions, or the IS element orientations. Our previous work showed that IS elements insert preferentially into the SIDD site (AT-rich and easy-to-open to form single strand DNA) that most likely overlaps with the upstream H-NS binding site [52]. H-NS preferentially binds to AT-rich and curved DNA regions. Thus, it is conceivable that insertions of IS elements completely or partially abolish H-NS binding, leading to various levels of *bgl* operon activation.

Positive regulators (suggested from previous studies) of the *bgl* operon, including Crp, BglJ, LeuO and RcsB, all of which bind to the *bgl* regulatory region, were used to reduce H-NS binding to the *bgl* regulatory region. First, strain G51 is essentially the same strain as G50 except for the presence of a point mutation at the Crp binding site, creating stronger Crp-cAMP binding to the *bgl* regulatory region (Fig. 3D) [6]. The operon activity of G51 showed a moderate increase compared to G50, although it is still Bgl^−^. To see if the increased operon activity was due to stronger Crp-cAMP binding to the *bgl* regulatory region, the *crp* gene was removed from the operon reporter G51-Z (G50-Z with a point mutation in the Crp operator), creating operon reporter Δ*crp*G51-Z. In the absence of *crp*, the β-galactosidase (LacZ) activity dropped to the level observed for G50-Z without the point mutation (Fig. S3). This confirmed that the actual stronger binding of Crp to the *bgl* regulatory region led to a substantial increase in the operon activity of strain G51-Z.

In addition, we overexpressed *bglJ*, *leuO* and *rcsB* in the background of G50, so that these positive regulators could (partially) outcompete H-NS binding to the *bgl* regulatory region (Fig. 3E). The operon activities increased compared to G50 when either *leuO* or *bglJ* was overexpressed individually (Fig. 3F). The operon activity doubled when *bglJ* and *rcsB* were overexpressed, but it dropped slightly when *rcsB* alone was overexpressed, as well as when *bglJ* was overexpressed in Δ*rcsB* cells. A similar trend was observed in the activity of operon reporter G50-Z when these positive regulators were overexpressed, indicating the validity of our results (Fig. S4). All of the strains showed Bgl^−^ phenotypes and were incapable of growing on salicin minimal agar plates. However, colonies of *leuO*, and *bglJ* overexpression strains appeared to be slightly red on MacConkey + salicin plates although these strains cannot utilize salicin as the sole carbon source. These growth phenotypes are consistent with low levels of the operon activity. These results indicate that over-production of LeuO, BglJ, RcsB or the BglJ/RcsB complex only partially decreased H-NS binding to the upstream site within the regulatory region.

### 2.4 H-NS binding within the *bglG* gene is necessary for *bgl* repression

**Fig. 4:**
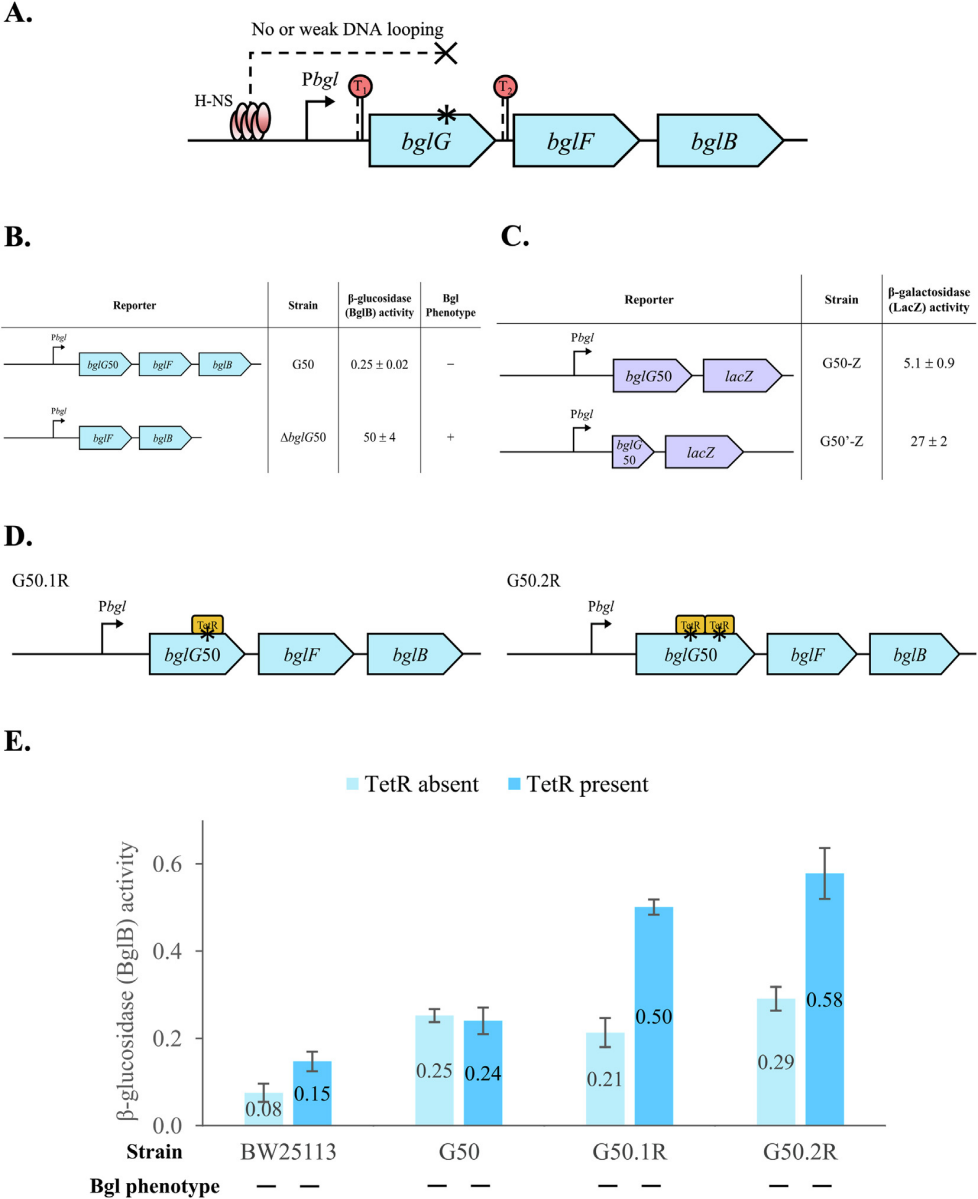
Breaking the DNA loop by preventing H-NS binding to its downstream site within the *bglG* gene. Strain G50 with deletion of both terminators flanking the *bglG* gene was used for these experiments. (A) Diagram showing that H-NS cannot bind to, or only weakly binds to, the downstream site within the *bglG* gene. Three approaches were used to prevent H-NS binding to the *bglG* site, including deletion of *bglG*50, deletion of a 3′-region of *bglG*50 and substitution of the proposed H-NS binding site(s) for one or two TetR binding sites. (B) Effects of completely deleting *bglG*50 on *bgl* operon expression. The left panel shows two operon reporters, strain G50 and Δ*bglG*50. The right panel shows the operon (BglB) activities and Bgl phenotypes of the two strains. (C) Effects of removing a 3′ region of *bglG*50 on *bgl* operon expression. The left panel represents two operon reporters. For strain G50′-Z, the 3′ region between +465 and +837 (relative to the *bglG* translation initiation site), thought to carry the H-NS binding site within *bglG*, was deleted. The right panel shows β-galactosidase (LacZ) activities, representing the *bgl* operon transcriptional activities. (D) Weakening H-NS binding by replacing the proposed H-NS binding region with one (left) or two (right) TetR operators. For strain G50.1R, a 19-bp DNA region between +544 and +564 (relative to the *bglG* translation initiation site) was replaced by one TetR operator sequence “tccctatcagtgatagaga”. For G50.2R, the 40-bp region between +523 and +564 was replaced by two identical TetR operators with sequences “tccctatcagtgatagagaCGtccctatcagtgatagaga”. See Material and Methods for details. (E) Effects of replacing the H-NS binding region with TetR operators within the *bglG* gene on *bgl* operon transcriptional activities, with or without TetR.

Previous studies had shown that the downstream H-NS binding site is approximately within positions +468 to +607, relative to the *bglG* translation initiation site [9]. We used several approaches to impede H-NS binding to this region, thus preventing the H-NS-mediated DNA looping (Fig. 4A). These approaches include: (1) deletion of the entire *bglG*50 region; (2) deletion of the 3′ region, thought to contain the H-NS binding site; and (3) substitutions of the H-NS binding region with one or two TetR operators.

The entire *bglG* gene was removed from G50 (Δ*bglG*50) to ensure that no binding of H-NS to *bglG* could occur. As shown in Fig. 4B, the operon activity of the resultant strain Δ*bglG*50 increased dramatically (200-fold) in comparison to G50, while Δ*bglG*50 shows a Bgl^+^ phenotype. These facts indicate that the H-NS binding site in the *bglG* gene is essential for *bgl* operon silencing by H-NS.

When a short version of *bglG*50, deleted for the 3′ region, from +466 to the end of the gene, was used for the operon *lacZ* reporter expression (that is, P*bgl*-*bglG*50′-*lacZ* in strain G50′-Z), an over 5-fold increase of operon activity was observed compared to G50-Z which carries the complete *bglG50* gene in front of the reporter (Fig. 4C). This fact suggests that the deleted 3′ region is important for H-NS repression of the *bgl* operon. Meanwhile, the smaller increase (5-fold increase for the truncated *bglG*50 versus 200-fold increase for the deletion of the entire *bglG*50 gene) may be due to the less effective binding of H-NS to the remaining region of the truncated *bglG* gene.

Strains G50.1R and G50.2R were created by replacing part of the putative H-NS binding site within the *bglG* gene with either one or two TetR operators, respectively (Fig. 4D). Both strains showed similar operon activities as for G50 when the TetR protein was absent, suggesting that such small region changes do not appreciably affect H-NS binding within the *bglG* gene. However, these strains showed a 2-fold increase in operon activities when the *tet* repressor TetR was expressed (Fig. 4E). These two strains showed Bgl^−^ phenotypes. Similar results were seen using operon reporters G50.1R-Z and G50.2R-Z (G50-Z with the H-NS binding site within *bglG* replaced by one or two TetR operators, respectively) (Fig. S5).

### 2.5 H-NS-mediated DNA looping within the *bgl* operon is DNA Phase-independent

**Fig. 5:**
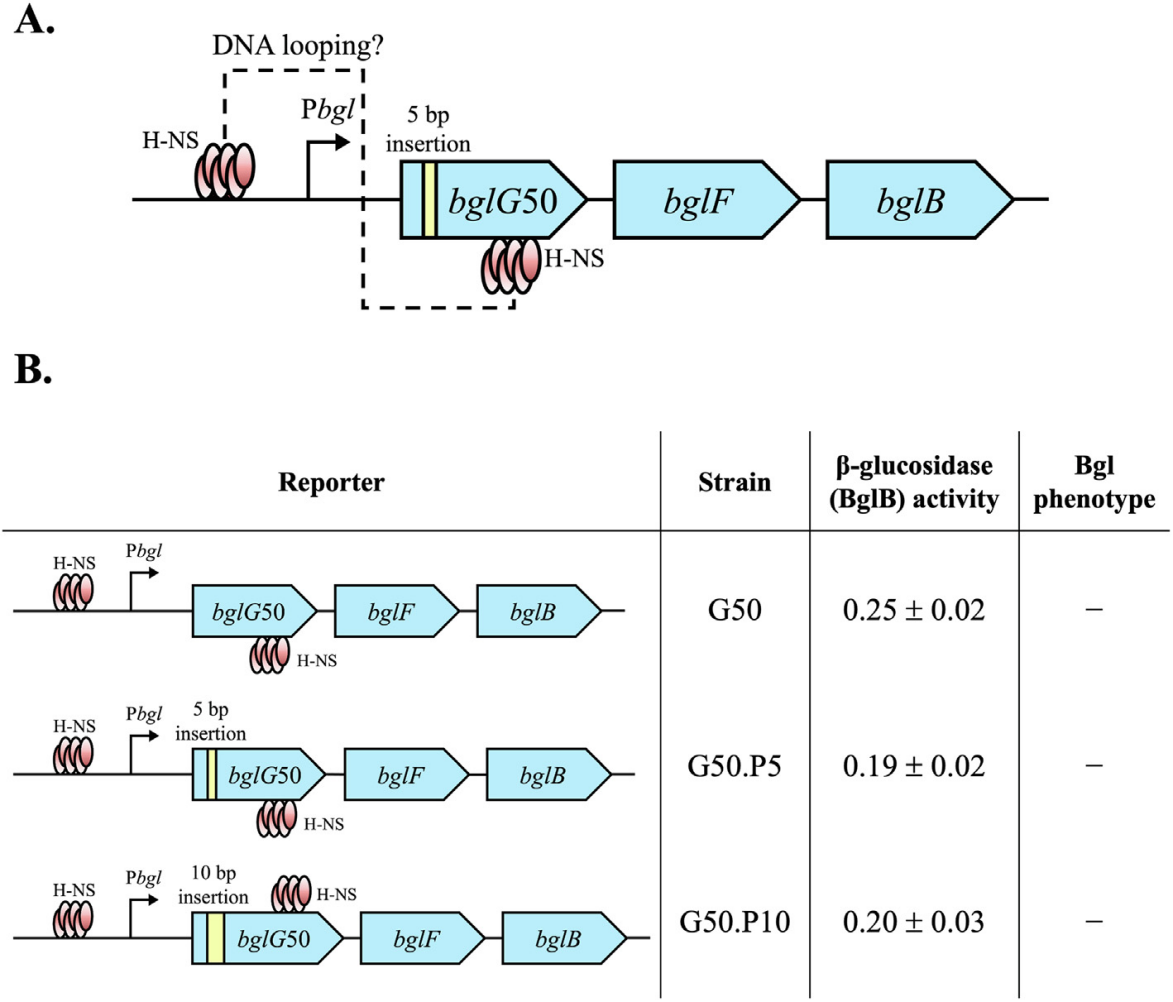
H-NS mediated DNA looping is DNA phase independent. (A) Diagram showing the change of DNA phasing between the upstream site and the downstream site by a 5-bp DNA insertion into the *bglG*50 gene. A 5-bp fragment (ATAGA) and a 10-bp fragment (ATAGAATAGA) were inserted individually into the *bglG* gene between +108 and +109 relative to the *bglG* translation initiation site in strain G50. (B) Effects of insertions of two small DNA fragments (5-bp and a 10-bp) within *bglG*50 on *bgl* operon expression. The left panel shows three operon reporters, with or without small DNA insertions within the *bglG*50 gene. The right panel shows the operon (assaying for BglB) activities and the Bgl phenotypes.

To investigate the characteristics of the putative H-NS loop, formed in the *bgl* operon, we first examined the impact of DNA phasing between the two H-NS binding sites on DNA loop formation (Fig. 5A). The DNA double helix is around 10.5 bp per turn. Two small DNA fragments of 5 bp and 10 bp were individually inserted within the *bglG* gene between the two H-NS binding sites, creating strains G50.P5 and G50.P10, respectively. As compared to strain G50, if DNA phasing between two binding sites were important, in contrast to strain G50.P10, repression would be diminished in strain G50.P5. Fig. 5B shows that these two strains had similar operon activities compared to G50, and they were still Bgl^−^. These results reveal that H-NS silencing of the *bgl* operon is DNA phase independent, and the DNA loop is stable.

### 2.6 H-NS-mediated DNA looping in the *bgl* operon is distance-independent

**Fig. 6:**
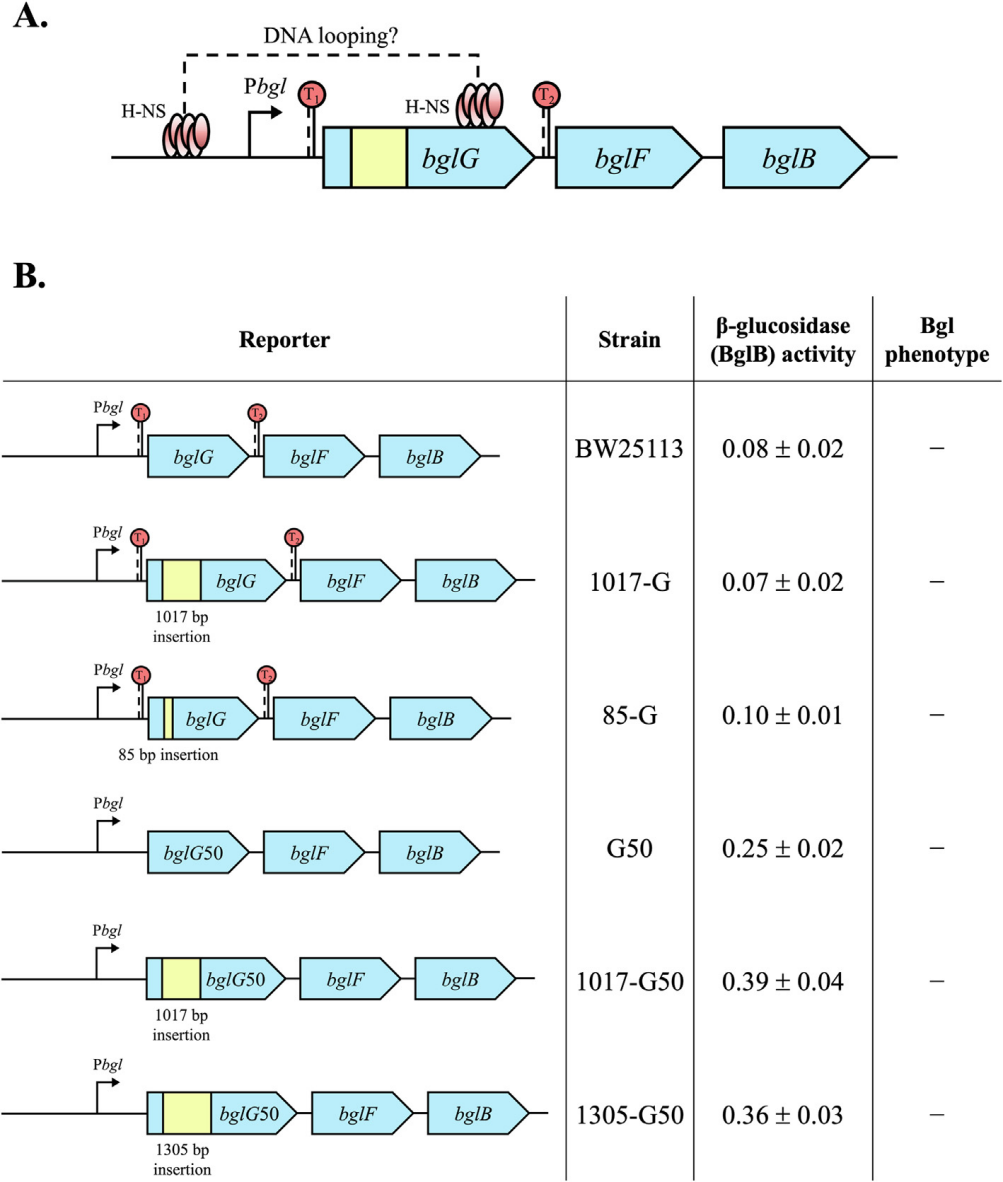
H-NS mediated DNA looping is independent of the length of intervening regions. (A) Diagram showing an insertion of a DNA fragment inside the *bglG* gene. A 1017 bp DNA fragment, carrying a *cat* gene flanked by two FRT sites, was inserted between two nucleotides, GA (+211 and +212 relative to the *bglG* translational initiation site), within the *bglG* gene. When the *cat* gene was flipped out, an 85-bp scar (the FRT site) was left. (B) Effects of increasing distances between the upstream site and the downstream site on *bgl* operon expression. The left panel shows the operon reporters with or without insertions of the two DNA fragments (1017 bp and 85 bp) into *bglG* or two fragments (1017 bp and 1305 bp) into *bglG*50. The right panel shows the operon activities and the Bgl phenotypes.

To further characterize H-NS-mediated DNA loop formation, we changed the distances between the two H-NS binding sites on the *bgl* operon (Fig. 6A). The distances were increased by individually inserting a 1017 bp DNA fragment and an 85 bp DNA fragment within *bglG* in the wild-type strain BW25113. These insertions were essentially without effect on *bgl* operon expression (Fig. 6B), suggesting that H-NS-mediated repression via the DNA loop is not affected by increased distances between two binding sites. However, these experiments could not rule out the possibility that the DNA fragment insertions inactivated the anti-terminator BglG, and that *bgl* operon transcription was terminated at each of the two terminators flanking *bglG*.

To eliminate a possible effect of one or both of the two terminators on DNA loop formation, we used strain G50 deleted for both terminators. As shown in Fig. 5B, the insertions of 5 bp and 10 bp DNA fragments between the upstream and the downstream binding sites within the *bglG*50 gene did not affect DNA looping. To further increase the distance, a 1017 bp fragment was inserted into *bglG*50 as well (1017-G50). As shown in Fig. 6B, this large insertion had a negligible effect on operon activity compared to G50. Similar results were observed when a 1305 bp fragment encoding Km resistance was inserted into the same location within the *bglG*50 gene. As expected, all of these modified strains with various sizes of DNA insertions within the *bglG* gene still showed Bgl^−^ phenotypes using our standard assays involving MacConkey + salicin plates and M9 + salicin plates.

To further confirm the absence of a terminator sequence within those two DNA fragments (1017 bp and 1305 bp) inserted at the *bglG*50 gene, which may otherwise abolish transcription of downstream *bglF* and *bglB*, the *hns* mutation was individually transferred to G50, 1017-G50 and 1305-G50 (Δ*hns*G50, Δ*hns*-1017-G50 and Δ*hns*-1305-G50). These new strains were examined for their BglB activities using our standard approach. As expected, a dramatically increased operon activity was seen for each strain, and there is little difference (10% or less) among these three new strains (Fig. S6), indicating that there is no terminator within each of these two DNA fragments inserted into *bglG*50. All of these results demonstrated that increasing distances between the two H-NS binding sites does not appreciably affect strong repression of the *bgl* operon by H-NS. This therefore indicates that H-NS-mediated DNA looping is robust.

## 3 Discussion

In this work, we have provided evidence that the nucleoid structuring protein, H-NS, silences the *bglGFB* operon using a DNA looping mechanism. As the major repressor, dimerized H-NS proteins have long been thought to bind to an upstream site within the regulatory region and a downstream site within the *bglG* gene. However, these bindings alone are not sufficient to abolish operon expression, although they do contribute to partial repression. Instead, by individually disrupting H-NS self-oligomerization or by preventing DNA binding to either of the two sites, we proposed that H-NS, when bound to both sites, bridges by loop formation the regulatory region and the *bglG* gene via oligomerization, forming the DNA loop and thus blocking transcription.

H-NS is characterized by self-dimerization and oligomerization via its *N*-terminal domain, leading to formation of higher-order oligomers [29], [30], [53], [54]. H-NS proteins exert their repressive effects on gene transcription by first binding to target DNA sites (usually AT-rich and curved DNA regions) through their C-terminal DNA-binding domains, forming nucleoprotein complexes. For stronger repression, as is responsible for gene silencing, H-NS oligomerizes along the DNA strand which either stiffens the DNA strand (forming a stiffened filament) or further forms a bridged nucleoprotein complex (a DNA loop) [33], [48], [55]. In relation to the *bgl* operon, H-NS is a strong dominant repressor which simultaneously binds to the *bgl* regulatory region and the 3′ region of the *bglG* gene [9]. H-NS binding to both locations is believed to be synergistic in repressing *bgl* operon expression, as the repression is much greater when H-NS binds to both locations than when it binds to either one [36].

**Fig. 7:**
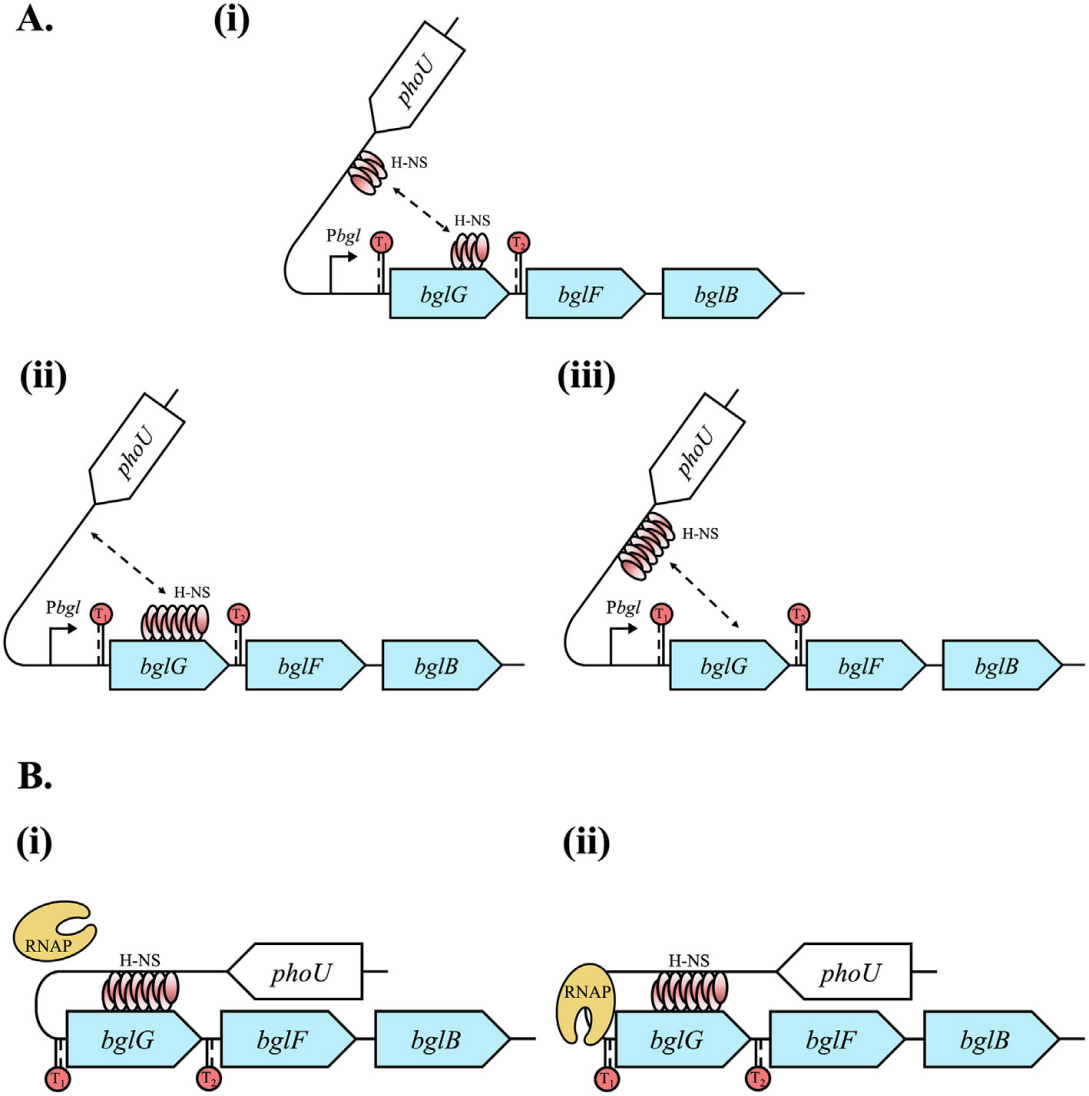
Model of *bgl* operon silencing by H-NS-mediated DNA looping. Each oval shape represents an H-NS monomer. The shades of color within the H-NS monomer represent the *N*-terminal oligomerization domain (red) and the C-terminal DNA binding domain (white). (A) H-NS binding and oligomerization properties lead to the formation of a stiffened nucleoprotein filament, either at (i) both or (ii-iii) one of its binding regions within the *bgl* operon. (B) The two H-NS binding regions, one at the *bgl* regulatory region and one within the *bglG* gene, are brought together, forming a DNA loop. (i) Upon the formation of a DNA loop, the RNA polymerase (RNAP) complex cannot access the promoter region, and therefore no transcription occurs. (ii) Alternatively, RNAP might still be able to bind to the *bgl* promoter (considering the relatively large DNA loop), but the polymerase is trapped inside the loop, therefore failing to transcribe mature *bglG* mRNA. This model is applicable to both the wild-type *bgl* operon (carrying both terminators) and the terminator-less *bgl* operon. (For interpretation of the references to color in this figure legend, the reader is referred to the web version of this article.)

In this work, we found that H-NS lost the vast majority (85%) of its repressive ability when it is deficient in self-oligomerization, even though it still maintains its DNA binding property. It is generally recognized that self-oligomerization is vital for H-NS to loop DNA [31], [33], [56], [57]. Furthermore, when binding to either of these two sites is disrupted or partially disrupted, H-NS repression is abolished or partially abolished, although it can oligomerize from one binding site. It is worthwhile to note that H-NSL30P fails to oligomerize due to perturbation of the dimerization domain (site 1) instead of its multimerization domain (site 2) [54]. Our results and previously published reports show that H-NSL30P is still capable of binding to DNA, thus repressing transcription [39]. Most likely, H-NSL30P proteins dimerize through the site 2, implying the formation of “non-canonical dimers”. Protein-protein interaction via site 1 is believed to be stronger than that via site 2. Based on these results, we propose a model by which H-NS silences the *bgl* operon (Fig. 7). Briefly, dimerized H-NS proteins first bind to both sites simultaneously or to either one site. Then DNA-bound H-NS dimers recruit more dimerized H-NS proteins extending along with the DNA strand(s), forming H-NS-DNA stiffened filaments. Subsequently, two binding sites are brought together to form a DNA loop. This may occur through binding of the C-terminal domain of each dimer within the filament to a nearby region of the second binding site (in the case of simultaneous binding to two locations) or directly to the region containing the second binding site (in the case of binding to one location). The entire *bgl* promoter and the 5’ region of *bglG* are embedded within the loop. Such an H-NS-mediated DNA loop efficiently abolishes the transcription of the *bgl* operon by (1) blocking the binding of RNA polymerase to the *bgl* promoter, and/or (2) trapping RNA polymerase when bound to the *bgl* promoter to prevent readthrough.

Instead of usually repressing canonical transcription, Singh et al. reported that H-NS primarily functions to repress spurious transcription of non-coding RNAs within the genes previously described as being H-NS repressed [58]. In the case of *bgl* operon regulation, in addition to the downstream binding site within *bglG*, there may be some other unidentified intragenic or intergenic promoters (for sigma70 to bind) overlapped with H-NS binding sites in both sense and antisense orientations. Transcription (leading to non-coding RNAs) from these promoters could be (strongly) repressed by H-NS as well. Although the possible presence of such new promoters and their repression by H-NS contribute to our understanding of the complex regulation of the *bgl* operon, these facts should not replace the main DNA looping mechanism by which H-NS silences the operon.

Similar to the *bgl* operon, the *proU* operon, encoding an osmoprotectant uptake system, is subject to strong H-NS repression as well [59], [60]. The repression requires H-NS binding to the regulatory region and a downstream region within the first gene *proV* of the operon [61]. The repression via these two sites is synergistic [36]. The distance between these two binding sites and their relative orientations on the face of the DNA helix had no major effect on *proU* repression by H-NS [62]. Combining all these reported findings, a looping mechanism similar to that in the *bgl* system would most likely be applicable to the *proU* operon.

Alterations of DNA phasing and the distances between two binding sites have negligible effects on H-NS-mediated looping, indicating that the formed DNA loop is exceptionally stable. This may be correlated to the large size (about 1 kb nucleotides) of the DNA loop, which confers rotational flexibility. The presence of such a robust DNA loop would definitely contribute to our understanding of the *bgl* operon as to its silencing state and its non-inducibility in wild-type cells. It is also conceivable that with such a stable DNA loop, involving the entire promoter region, other transcription factors, such as BglJ, RcsB, LeuO and Crp, when expressed at the wild-type levels, either fail to access or are trapped in the promoter region, thus being essentially without effects on *bgl* operon expression. When these proteins are produced in large amounts or have better binding sites, the binding of H-NS to the upstream site may be partially prevented, thereby elevating rates of transcription.

Indicator agar plates such as those containing MacConkey agar with salicin or bromothymol blue with salicin have been used to determine the phenotypes of growth/fermentation on/of β-glucosides, with a dark color (red or blue) developed within individual colonies that are Bgl^+^ (able to utilize β-glucoside for growth) [7], [11]. However, the color changes of colonies on these indicator plates do not always represent the actual capability of the cells to grow on β-glucosides as the sole carbon source, as all cells are able to grow on the indicator plates. In fact, some mutant strains that were claimed to be Bgl^+^ in previous studies, were not able to grow on minimal agar plates with salicin as the sole carbon source. In this report, we show that overproduction of BglJ, RcsB, the BglJ/RcsB complex or LeuO did not give rise to appreciable growth on minimal salicin agar media although visible red color could be seen at the centers of some of their colonies on MacConkey agar plates containing salicin. Therefore, phenotypic results obtained from MacConkey indicator plates with salicin were sometimes misleading. As a result, strains considered to have a Bgl^+^ phenotype in this study must fulfill two requirements: (1) appearance of colonies with visible red color on MacConkey agar plates containing 0.5% salicin after 1 day of incubation at 37°C, and (2) appearance of colonies of visible sizes on M9 agar plates with salicin as the sole source of carbon after 2 days of incubation at 37°C.

Flanking the *bglG* gene are two terminators that effectively block transcription due to the formation of hairpin-like secondary structures in the absence of the activated antiterminator, BglG [12]. It was of interest to know if these transcriptional terminators contribute to *bgl* operon silencing. Using a mutant strain (G50) only deleted for these two terminators with all other regions unchanged, we showed that its operon activity increased only slightly compared to the wild-type, and it showed a Bgl^−^ phenotype (Fig. 1B). This indicated that the two terminators flanking the two sides of *bglG* do not exert an appreciable effect on the silencing of the *bgl* operon in wild-type *E. coli* cells. We thus distinguished the roles of H-NS and both terminators with respect to their regulatory effects on *bgl* operon expression. Strain G50 (without the two *bgl* terminators) was used throughout this study, showing that H-NS is still able to form a DNA loop, thus silencing *bgl* operon expression, when the terminator structures are not present.

Overexpression of positive regulators, BglJ and LeuO, increased *bgl* operon transcriptional activities under H-NS repression (Fig. 3F). This is consistent with the notion that reducing H-NS binding to the promoter region can (partially) eliminate DNA looping, thereby enhancing operon transcription. However, this is not the case for another positive regulator, RcsB, as its overproduction did not appreciably affect operon expression activity (columns 3 and 4 in Fig. 3F). As BglJ and RcsB form heterodimers when regulating *bgl* operon expression [11], BglJ might be the limiting factor with RcsB present in normal (wild type) amounts. The expression of *bglJ* is strongly repressed by H-NS, leading to very low levels of BglJ in wild-type *E. coli* cells [11], [63]. This explains our results showing that *bgl* operon expression was increased when *bglJ* was overexpressed but not when *rcsB* was overexpressed. Interestingly, even though the operon activity increased when both *bglJ* and *rcsB* were overexpressed simultaneously, the increased level was not as great as that when *bglJ* was overexpressed alone (columns 3 and 5 in Fig. 3F). Similar trends were obtained when using either β-glucosidase (BglB) or β-galactosidase (LacZ) assays, substantiating the validity of the data. At the same time, a growth defect has been observed for the cells when *rcsB* was overexpressed (data not shown). As RcsB is a global regulator, overexpression of *rcsB* might have caused a pleiotropic effect on transcription of other genes and cell growth in addition to affecting *bgl* operon transcription [64].

A moderate increase of *bgl* operon expression was seen when stronger Crp-cAMP binding to the *bgl* regulatory region occurred. However, all strains, either producing higher levels of positive regulators or carrying a better binding site, are still Bgl^−^ due to the low levels of operon expression (Fig. 3D and F). Most likely, these regulators cannot efficiently bind to their operators within the regulatory region due either to an insufficient affinity, or to the presence of a stable DNA loop formed by H-NS. It would be interesting to know how these regulators at elevated levels or with better operators affect *bgl* operon transcription in the absence of H-NS although there was no effect on *bgl* operon transcription when the gene for any one of these factors was deleted in the absence of H-NS [39].

Previous studies had reported that H-NS binds to a region approximately 600 to 700 base pairs downstream of the *bgl* promoter, which is around positions +468 to +607 relative to the *bglG* translational initiation site [9]. In this study, we replaced part of this region, presumably the H-NS binding site, within the *bglG* gene by one or two TetR operators, resulting in small increases in *bgl* operon activity when compared to the control (Fig. 4E). However, the operon activities increased even more when TetR was present. These results suggest that: (1) the binding of TetR to the *bglG* gene only partially or occasionally decreased H-NS binding to its target site with the *bglG* gene; (2) the binding of TetR to two operators within the *bglG* gene further impeded H-NS binding to its target site, reducing the H-NS looping ability and increasing transcription, even though the bound TetR might act as a roadblock, blocking transcription; and (3) the region replaced by the TetR binding sites might not be the actual H-NS binding site within the *bglG* gene, or H-NS might be able to bind to (an)other location(s) in the gene as deletions of the entire *bglG* gene or a larger portion of the gene either eliminated or substantially disrupted DNA looping.

Our recent work revealed that the frequency of Bgl^+^ mutations mediated by small IS transposons are positively regulated by the level of the anti-terminator protein, BglG [51]. It will be most interesting to investigate: (1) whether and how H-NS or H-NS-mediated DNA looping is involved in affecting IS insertional mutations; and (2) whether and how H-NS binding affects the SIDD (Superhelical stress-Induced DNA Duplex Destabilization) structure [52] in the regulatory region. Further studies should be also carried out to examine interactions between the two H-NS bound regions within the *bgl* operon using the 3C technology [65] and to directly visualize (and measure) H-NS-mediated DNA looping within the *bgl* operon in the various types of live *E. coli* cells used in this study [66]. Such investigations should contribute to our understanding of the looping structure and its dynamics.

## 4 Materials & Methods

### 4.1 *E. coli* strains

*E. coli* K12 strain BW25113 [67] was used as the parental strain that is referred to as “wild-type” strain in this study. All other strains are derived from this strain. BW-RI is another wild-type strain used in this study, which constitutively produces the TetR protein, the repressor of the *tet* promoter, P*tet* [68].

### 4.2 Construction of an intermediate strain, ΔP*bglG*

Using wild type strain BW25113, the chromosomal region containing the *bgl* promoter (P*bgl*) and the *bglG* gene plus the *bglG*-flanking terminators was replaced by a *cat* gene amplified from pKD3 [67] by oligos Pbglcat-P1 and bglFcat-P2 (Table S2). The *cat* gene was flipped out by pCP20, yielding strain ΔP*bglG*, in which both P*bgl* and *bglG* with the two terminators were replaced by an 85-bp FRT scar. This strain was then used as a beginning strain to make several other strains.

### 4.3 Removal of two terminators flanking the *bglG* gene

The first gene in the *bgl* operon, *bglG*, is flanked by two transcriptional terminators. To determine if these terminators are important for H-NS mediated *bgl* operon silencing, they were deleted from the chromosome. To do this, the strain ΔP*bglG* was used as the starting strain. Present in plasmid pKES50 [69], a P*bgl*-*bglG*50-*lacZ* cassette consisted of the *lacZ* gene (with its own Shine-Dalgarno sequence) fused to a region downstream of the *bgl* promoter (P*bgl*), a modified *bglG* gene, *bglG*50, (note that this modified gene has no flanking terminators, and it is not translated because the first two ATG codons were changed to GCG). The P*bgl*-*bglG*50 DNA region together with the downstream *lacZ*’s RBS (between *bglG*50 and *lacZ*) was amplified from plasmid pKES50 using oligos Pbgl-Xh-Fbg and bglG-Bm-Rm (Table S2). The PCR products were gel purified, digested with *Xho*I and *BamH*I, and then ligated into the same sites of pKDT [70], where “T” refers to the *rrnB* terminator (*rrnB*T). This yielded pKDT_P*bgl*-G50, in which the native *bgl* promoter drives the terminator-less *bglG* gene (*bglG*50). The cassette containing the *km*^r^ gene, *rrnB*T and P*bgl*-G50 (*km*^r^:*rrnB*T:P*bgl*-G50) was amplified from pKDT_P*bgl*-G50 using oligos Pbgl.G50-P1 and bglF.G50-P2 (Table S2). The PCR products were gel purified and electroporated into ΔP*bglG* cells expressing the Lambda Red proteins to replace the 85-bp FRT scar. This yielded strain G50, in which the native *bgl* promoter drives a terminator-less *bglG* gene (*bglG*50) followed by *bglF* and *bglB*. The intergenic region (aatagcttcacaggaaacagct) between *bglG* and *bglF* is the same as one between *bglG* and *lacZ* in pKES50 [69], and it carries the *lacZ*’s RBS for *bglF*.

The strain G50 was used as the recipient strain for P1 transduction in order to make other strains. First, the transcription unit including a constitutively expressed *tetR* gene and a spectinomycin resistance (*sp*^r^) marker was transferred to G50 by P1 transduction as mentioned above, yielding strain RIG50. Also, the deletion of *hns* was transferred to G50 by P1 transduction, yielding Δ*hns*G50.

### 4.4 Removal of the presumptive H-NS binding site within the *bgl* regulatory region

H-NS binds to two locations in the *bgl* operon, one within the *bgl* regulatory region and the other within the *bglG* gene, although exact binding sites have not been defined [9], [10]. To abolish or reduce H-NS binding to the *bgl* regulatory region, the upstream region (located between positions −585 to −207 relative to the *bglG* translational initiation site) carrying the possible H-NS binding site in G50, was first replaced by a *km*^r^ gene amplified from pKD4 using oligos Pbglcat-P1 and Pbgl2-P2 (Table S2). The *km*^r^ gene was then flipped out by pCP20, yielding strain G50ΔO_HNS_-P*bgl*.

### 4.5 Screening IS insertional Bgl^+^ mutants

Insertion of IS elements is believed to abolish or reduce H-NS binding to the *bgl* regulatory region. To obtain IS insertional mutants, wild-type BW25113 cells were grown in LB medium at 30°C for at least 6 hrs. The cells were washed twice with M9 salts with no carbon source, and around 10^8^ cells were inoculated onto M9 or M63 minimal agar plates containing 0.5% salicin as indicated below. The plates were incubated at 30°C and were examined daily for the appearance of Bgl^+^ colonies [51]. The colonies were subject to PCR analyses using Pbgl-F2 and Pbgl-R2 (Table S2) to verify the presence of any insertional mutations at the *bgl* regulatory region. The type, location, and orientation of IS elements inserted within the *bgl* regulatory region were identified by DNA sequencing.

The IS insertional mutant, IS5r with IS5 inserted in reverse orientation at −207.5 relative to the *bglG* translational initiation site, was used as the positive control. The deletion of *hns* was transferred to IS5r by P1 transduction, yielding strain IS5rΔ*hns*.

### 4.6 Construction of strain G51 with a stronger Crp-cAMP binding site at P*bgl*

To make strain G51, plasmid pKES51 [69] was used as the template to construct plasmid pKDT_P*bgl*-G51, which is essentially the same as pKDT_P*bgl*-G50 except that the presence of a stronger Crp-cAMP operator (O*_Crp_) in P*bgl* resulted from a C to T substitution at position −134 relative to the *bglG* translational initiation site [6]. Using similar methods as described above for G50, the cassette of P*bgl*_O*_Crp_, driving *bglG* expression, was integrated upstream of *bglF* in ΔP*bglG* cells, yielding strain G51. This strain is the same as G50 except that it carries a stronger Crp-cAMP binding site in the *bgl* promoter region.

### 4.7 Construction of P*tet* driving *bglJ*, *rcsB*, and *leuO* on the chromosome

Using plasmid pKDT:P*tet* [70] as a template, the cassette “*km*^r^:*rrnB*T:P*tet*”, containing the *km*^r^ gene, the *rrnB* terminator (*rrnB*T) and the P*tet* promoter, was amplified using the primer pair Ptet-bglJ-P1 and Ptet-bglJ-P2 (Table S2). The PCR products were integrated into the chromosome of strain G50 (without the two *bgl* terminators) to replace the *yjjQ* promoter (from −73 to +1 relative to the *yjjQ* translational start point). Chromosomal integration was confirmed, first by colony PCR, and subsequently by DNA sequencing. This yielded strain G50P*tet*-*bglJ*, in which the strong *tet* promoter drives the *yjjQ*-*bglJ* operon. Similarly, the “*km*^r^:*rrnB*T:P*tet*” cassette amplified by Ptet-leuO-P1 and Ptet-leuO-P2 (Table S2) from pKDT:P*tet* was substituted for the *leuO* promoter region (from −151 to −1 relative to the *leuO* translational start point), yielding strain G50P*tet*-*leuO*. The same cassette amplified by Ptet-rcsB-P1 and Ptet-rcsB-P2 (Table S2) was substituted for the *rcsD*/*rcsB* intergenic region (from −12 to −1 relative to the *rcsB* translational start point), yielding strain G50P*tet*-*rcsB*.

To make a *bglJ*/*rcsB* double overexpression strain, the *km*^r^ gene was first flipped out from G50P*tet*-*bglJ* by pCP20, yielding G50P*tet*-*bglJ*-*km*^s^, which is sensitive to kanamycin. The *rcsB* gene, driven by the strong *tet* promoter with a *km*^r^ marker, was then transferred to G50P*tet*-*bglJ*-*km*^s^ by P1 transduction, yielding strain G50P*tet*-*bglJ*/*rcsB.* The *rcsB* mutation was transferred to G50P*tet*-*bglJ*-*km*^s^ as well, yielding strain G50P*tet*-*bglJ*Δ*rcsB*, in which the strong *tet* promoter drives *bglJ* in the absence of *rcsB*.

### 4.8 Removal of the entire *bglG* gene

To completely abolish H-NS binding in the *bglG* gene, the entire *bglG* gene and its two flanked terminators were deleted. First, the region from −78 to +946 relative to the *bglG* translational initiation site, containing the first terminator, the *bglG* gene and the second terminator, was replaced by a *km*^r^ gene amplified from pKD4 [67] using oligos G50-P1 and G50-P2 (Table S2). The *km*^r^ gene was then flipped out using pCP20, yielding strain Δ*bglG*50, in which the *bglG* gene with the two flanking terminators was replaced by an 85 bp fragment.

### 4.9 Replacing the proposed H-NS binding site within the *bglG* gene with one or two TetR Operator(s)

In vitro protein/DNA binding assays showed that H-NS proteins are capable of binding to a region within the *bglG* gene, which is located at +479 to +606 relative to the *bglG* translational initiation site [9]. To reduce H-NS binding to this region (the presumptive H-NS binding site), part of this region in strain G50 was replaced by one or two operators of TetR using the TetA-SacB positive and negative selection system [71]. Briefly, to add one TetR operator, the 19 nucleotides with sequence “aaatgctgcaattaataaa”, between +544 and +564 relative to the *bglG* translational initiation site, was replaced by one TetR operator with sequence “tccctatcagtgatagaga” [72], yielding G50.1R. Similarly, a region of 40 nucleotides with sequence “gtgtcacgcagttaatgcgcgaaatgctgcaattaataaa” (between +523 and +564) was replaced by two identical TetR operators with sequences “tccctatcagtgatagagaCGtccctatcagtgatagaga” (two TetR operators separated by two nucleotides CG, in the center), which yielded G50.2R. The *tetR*-*sp*^r^ cassette (constitutively expressing *tetR*) was transferred from BW-RI to G50.1R and G50.2R, yielding strains RIG50.1R and RIG50.2R, respectively.

### 4.10 Insertions of 5 bp and 10 bp fragments between the two H-NS binding sites

DNA is a double-stranded helix with 10–10.5 bps per turn. To determine if DNA looping within the *bgl* operon, formed via H-NS protein oligomerization, is DNA phase-dependent, a 5-bp fragment with nucleotides “ATAGA” and a 10-bp fragment with nucleotides “ATAGAATAGA” were inserted individually into the *bglG* gene between +108 and +109 relative to the *bglG* translational initiation site in strain G50. Briefly, these two small fragments (5 bp and 10 bp) were first integrated into the designated locus (between +108 and +109) within the *bglG*50 gene in plasmid pKDT_P*bgl*-G50 using fusion PCR, yielding plasmids pKDT_P*bgl*-G50.5 bp and pKDT_P*bgl*-G50.10 bp, respectively. Using these plasmids as templates, the “*km*^r^:*rrnB*T:P*bgl*-G50.P5” cassette and the “*km*^r^:*rrnB*T:P*bgl*-G50.P10” cassette were amplified using oligos Pbgl.G50-P1 and bglF.G50-P2 (Table S2). The PCR products were electroporated into ΔP*bglG* cells to substitute for the 85-bp scar that replaced the *bgl* regulatory region and the *bglG* gene together with the two flanking terminators. Several Km resistant colonies were confirmed by colony PCR and DNA sequencing to carry a 5-bp insertion or a 10-bp insertion at the expected location within the *bglG* gene, yielding strains G50.P5 and G50.P10. Compared to the original G50 strain, the phasing of those two H-NS binding sites is almost unchanged in strain G50.P10. However, the phasing should be essentially opposite in strain G50.P5.

### 4.11 Changing the distance between the two H-NS binding sites

To determine if the stability of H-NS-mediated looping in the *bgl* operon is size dependent, the distance between the two H-NS binding sites was increased. A 1017-bp fragment carrying a *cat* gene from pKD3 was inserted into the beginning of the *bglG* gene of BW25113, between +209 and +210, relative to the *bglG* translational initiation site. In the resulting strain, the 1017-bp DNA fragment carrying the *cat* gene with its own promoter and two FRT sites were inserted into the *bglG* gene between the two H-NS binding sites, oriented in an opposite direction as the *bglG* gene, yielding strain 1017-G. The *cat* gene was then flipped out using pCP20, yielding strain 85-G, in which an 85-bp fragment is left between the two H-NS binding sites. Similarly, a *cat* gene was inserted into the same location of the *bglG*50 gene in the G50 background, yielding 1017-G50, with a 1017 bp distance increase between the two H-NS binding sites. Further, a *km*^r^ gene (1305 bp), encoding kanamycin resistance, was amplified from pKD13 and subsequently inserted into the same location as for 1017-G50, yielding 1305-G50 that carries a 1305 bp fragment between the two H-NS binding sites.

The two strains, 1017-G50 and 1305-G50, were used as the recipient strains for transferring the deletion of *hns* by P1 transduction, yielding strains Δ*hns*-1017-G50 and Δ*hns*-1305-G50.

### 4.12 Construction of the *bgl* operon LacZ reporter

Strain BW25113_Z [51] carries a *bgl* operon LacZ reporter, which is composed of the *bglGFB* promoter, the first terminator, *bglG*, the *bglG*/*bglF* intergenic region (with the second terminator) and the first 9 codons of *bglF*, plus a stop codon at the 3′ end, immediately upstream of the *lacZ*’s RBS at the *lac* locus. A *km*^r^ gene followed by a *rrnB* terminator is located upstream of this reporter. Using the same protocol for strain BW25113_Z construction [51], the cassettes of “*km*^r^:*rrnB*T:P*bgl*-*bglG*50” on plasmid pKDT_P*bgl*-G50 and “*km*^r^:*rrnB*T:P*bgl*-*bglG*51” on plasmid pKDT_P*bgl*-G51 were individually integrated into the chromosome of the default strain MGIBKY [70] to replace the *lacI* gene and the *lacI*/*lacZ* intergenic region, yielding strains G50-Z and G51-Z. These two reporters were transferred into a Δ*crp* Glp^+^ mutant (able to grow on glycerol) [73], yielding Δ*crp*G50-Z and Δ*crp*G51-Z, respectively.

To determine the effects of overexpression of positive regulators on H-NS binding to the upstream site within the regulatory region, the P*bgl*-*bglG*50-*lacZ* operon reporter was transferred from G50-Z to strains G50P*tet*-*bglJ*, G50P*tet*-*leuO*, G50P*tet*-*rcsB*, G50P*tet*-*bglJ*/*rcsB* and G50P*tet*-*bglJ*Δ*rcsB*, yielding G50-Z-P*tet*-*bglJ*, G50-Z-P*tet*-*leuO*, G50-Z-P*tet*-*rcsB* G50-Z-Ptet-*bglJ*/*rcsB* and G50-Z-P*tet*-*bglJ*Δ*rcsB*, respectively.

As described above, H-NS proteins are capable of binding to a region within the *bglG* gene, which is located between +479 and +606 relative to the translational initiation site of *bglG*. To examine the effect of this region on β-galactosidase (LacZ) activity, part of this region of the *bglG*50 gene in plasmid pKDT_P*bgl*-G50 was replaced by one or two operators of TetR using fusion PCR, yielding pKDT_P*bgl*-G50.1R and pKDT_P*bgl*-G50.2R, respectively. The cassette of “*km*^r^:*rrnB*T:P*bgl*-*bglG*50.1R” on pKDT_P*bgl*-G50.1R and the cassette of “*km*^r^:*rrnB*T:P*bgl*-*bglG*50.2R” on pKDT_P*bgl*-G50.2R were individually transferred to the same location on the chromosome as for G50-Z, yielding strains G50.1R-Z and G50.2R-Z, respectively. The two operon reporters are the same as G50-Z, except that part of the H-NS binding site within *bglG* was replaced by one or two TetR operators. Using G50.1R-Z and G50.2R-Z as recipient strains, two new operon reporters were made by transferring the *tetR* expression unit with a *sp*^r^ marker using P1 transduction, yielding RIG50.1R-Z and RIG50.2R-Z, respectively.

To further prevent H-NS binding to *bglG*50, a new operon reporter similar to G50-Z, carrying the *bgl* promoter and only the 5′ region of *bglG*50 with no 3′ region, was made. The cassette of “*km*^r^:*rrnB*T:P*bgl*-*bglG*50’” (*bglG*50’ refers to the region from +1 to +465 relative to the *bglG* translational initiation site), amplified from pKDT_P*bgl*-G50 by oligos P1n-P1n and G50tn-Z-P2 (Table S2), was fused to the *lacZ* gene as for other operon reporters, yielding operon reporter G50’-Z. There is a stop codon (TAA) added to the end of *bglG*50’, and the RBS for *lacZ* remains the same as for G50-Z.

To determine if H-NS or H-NSL30P has an inhibitory effect on expression of *bglG* when bound to it, another new *lacZ* transcriptional reporter was made in which the *bglG*-*lacZ* fusion is driven by a constitutive promoter. The cassette, “*km*^r^:*rrnB*T:Iq-*bglG*” (with *lacIq* driving *bglG* expression), was amplified from strain Iq-G [51] and subsequently fused upstream of *lacZ* as for other operon *lacZ* reporters at the *lac* locus. This reporter, carrying a *lacIq* constitutive promoter driving the terminator-less *bglG* gene that is transcriptionally fused to a *lacZ* gene, was transferred into strains Δ*hns* and *hns*L30p, yielding Δ*hns*_Iq-G-Z and *hns*L30P_Iq-G-Z, respectively. Similarly, the cassette “*km*^r^:*rrnB*T:P*tet*” from strain P*tet*-G [51] was fused upstream of *lacZ* and subsequently transferred to Δ*hns* and *hns*L30P, yielding Δ*hns*_P*tet*-G-Z and *hns*L30P_P*tet*-G-Z, respectively.

### 4.13 Determining the Bgl^+^/Bgl^−^ phenotypes of strains

*E. coli* cells were grown on MacConkey indicator plates with 0.5% salicin at 37°C overnight. Visible red color was shown for colonies with a Bgl^+^ phenotype, indicating their ability to ferment salicin. Otherwise, colonies with a Bgl^−^ phenotype remained white in color. In addition, cells were grown on M9 minimal agar plates with 0.5% salicin as the sole carbon source at 37°C for 2 days to further confirm their phenotype. To have a Bgl^+^ phenotype, cells should form visible colonies during the 2-day incubation. Otherwise, the cells are considered to be Bgl^−^. In this study, we define a Bgl^+^ phenotype as the appearance of red colonies on MacConkey + salicin plates during a 24-hr incubation plus the formation of visible colonies on M9 + salicin plates during a 48-hr incubation.

### 4.14 β-Glucosidase (BglB) assay

The protocols for β-glucosidase assay as described in previous studies were followed with minor changes [12], [74]. Briefly, the cells were grown in LB medium at 37°C with shaking. After at least 6 hrs, 20 µL of the culture were transferred to 1x M63 minimal medium with 0.66% casamino acids; then it was left overnight for growth at 37°C with shaking. An appropriate amount of the overnight culture was inoculated into 6 mL of M63 minimal medium with 0.66% casamino acids and 0.5% salicin, at a starting OD_600_ of 0.025. The cells were grown at 37°C with shaking. Five samples with 0.8 mL each were collected during the late exponential growth phase when the *bgl* operon was fully induced with an OD_600_ of above 1.5. The samples were centrifuged at a speed of 5,500 rpm for 2.5 mins. The supernatant was removed, and the cells were suspended with 1 mL of Z-buffer with 50 µg/mL chloramphenicol.

The samples were warmed in a 37°C water bath. To start the reaction, 200 µL of *p*-nitrophenyl-β-d-glucoside (PNPG, 8 mg/mL) were added to the cell suspension in Z-buffer. After a visible yellow color appeared, 0.5 mL of 1 M sodium carbonate was added and vortexed to stop the reaction. The reaction mixture was diluted, if appropriate, and centrifuged at a speed of 15,000 rpm for 2.5 mins. The absorbance of the reaction mixture was measured at 420 nm and 550 nm. The BglB activity of each sample was calculated using the equation: β-glucosidase (BglB) activity = [1000 × (OD_420nm_ – 1.75 × OD_550nm_) × Dilution factor]/[OD_600nm_ × Time of reaction (min) × Volume of sample (mL)]. The activity of the strain was determined by averaging the BglB activities of the samples measured.

### 4.15 β-Galactosidase (LacZ) assay

Reporter strains were grown in LB medium at 37°C with shaking. After at least 6 hrs, 20 µL of the culture were transferred to 3 mL of M63 minimal media with 0.5% glycerol. Then it was left overnight for growth at 37°C with shaking. The overnight culture was inoculated into 5 mL of M63 minimal medium with 0.5% glycerol, with a starting OD_600_ of 0.03. The cells were grown at 37°C with shaking. Four samples were collected during the exponential growth phase, when the OD_600_ reaches around 0.2, 0.4, 0.6 and 0.8. Collected samples could be kept frozen at −20°C prior to the measurements.

200 µL of samples and 800 µL of Z-buffer were mixed with 25 µL of chloroform by vortexing for 10 s twice. The samples were warmed in a 37°C water bath. To start the reaction, 200 µL of *o*-nitrophenyl-β-d-galactopyranoside (β-ONPG) was added to each sample. After a visible yellow color appeared in some tubes, 0.5 mL of 1 M sodium carbonate was added and vortexed to stop the reaction. The reaction mixtures were diluted, if appropriate, and centrifuged at a speed of 15,000 rpm for 2.5 mins. The absorbance of the reaction mixture was measured at 420 nm and 550 nm. The LacZ activity of each sample was calculated using the equation [75]: β-galactosidase (LacZ) activity (Miller units) = [1000 × (OD_420nm_ – 1.75 × OD_550nm_) × Dilution factor]/[Time of reaction (min) × Volume of sample (mL)]. The activity of the strain was determined by the slope of the LacZ activities versus cell densities (OD_600_) of the four samples collected at different times.

## CRediT authorship contribution statement

**Katie Jing Kay Lam:** Methodology, Investigation, Data curation, Writing – original draft, Writing – review & editing, Visualization. **Zhongge Zhang:** Conceptualization, Methodology, Data curation, Writing – original draft, Writing – review & editing, Supervision. **Milton H. Saier:** Conceptualization, Writing – review & editing, Supervision, Funding acquisition.

## Declaration of Competing Interest

The authors declare that they have no known competing financial interests or personal relationships that could have appeared to influence the work reported in this paper.
