# Supplementary material for: Histone-like nucleoid structuring (H-NS) protein silences the beta-glucoside (bgl) utilization operon in Escherichia coli by forming a DNA loop
Source: Comput Struct Biotechnol J. 2022 Nov 12;20:6287–301. doi: 10.1016/j.csbj.2022.11.027 (PMC9678765; doi:10.1016/j.csbj.2022.11.027)
Supplement: Supplementary data 1 [file mmc1.docx]

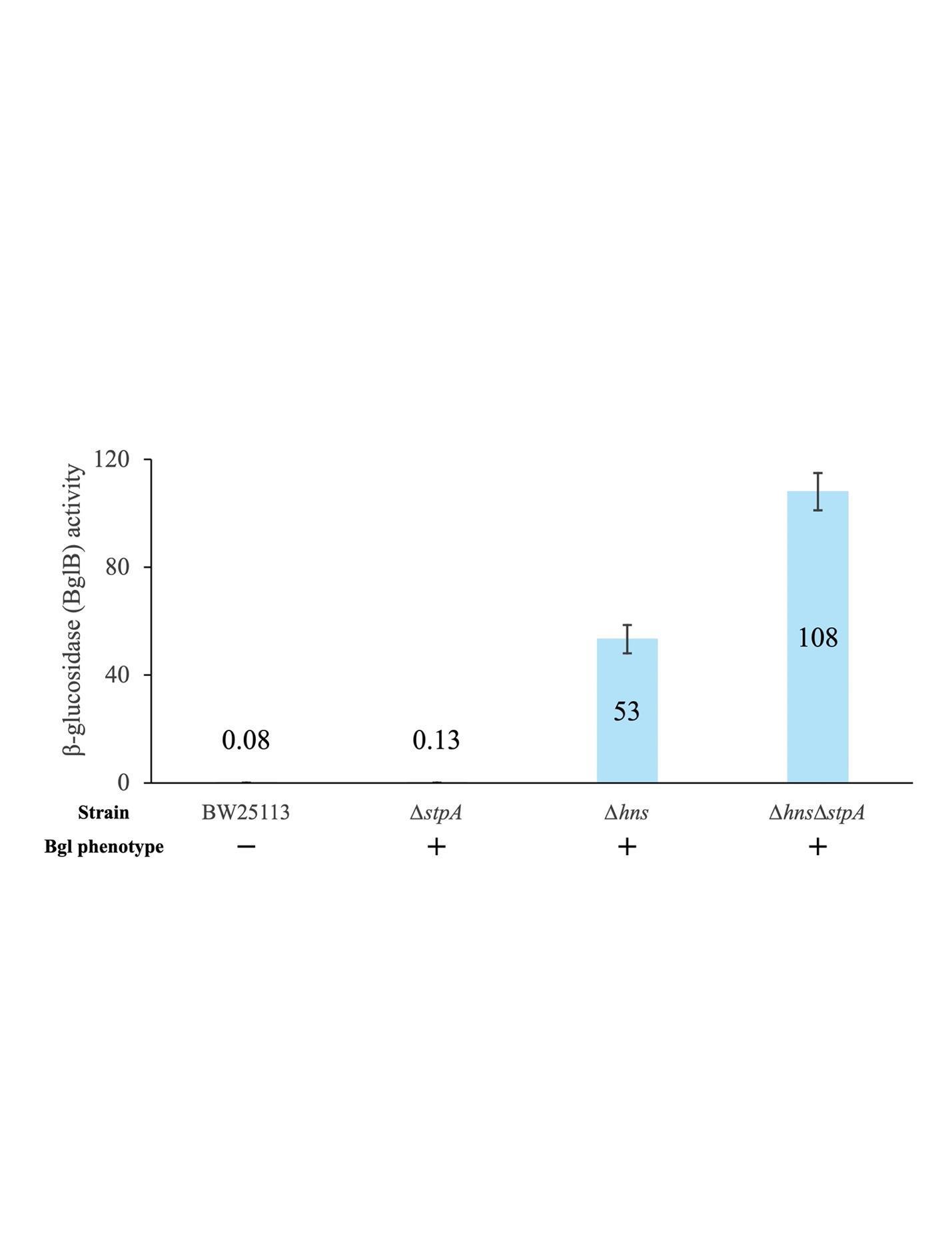


**Figure S1. StpA represses *bgl* operon expression in the absence of H-NS.** The β-glucosidase (BglB) activities and Bgl phenotypes of strains BW25113, ∆*stpA*, ∆*hns* and ∆*hns*∆*stpA* were determined (see Materials and Methods).


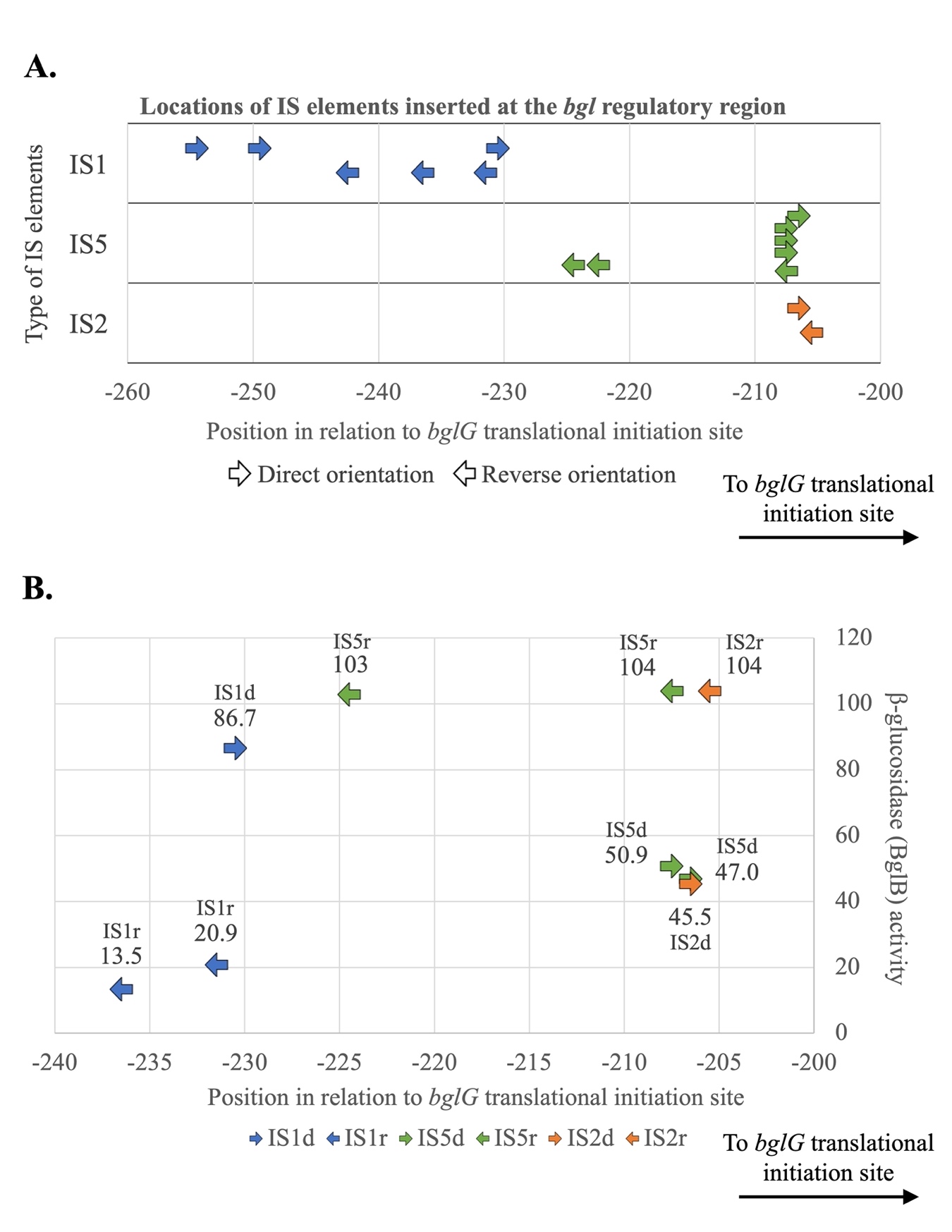


**Figure S2. Chromosomal locations and orientations of IS elements inserted in the *bgl* regulatory region and their effects on *bgl* operon expression.** (**A**) Locations and orientations of IS1, IS2 and IS5 elements upstream of the *bgl* regulatory region. **(B**) Effects of insertions of IS1, IS2 and IS5 in both orientations on *bgl* operon expression.


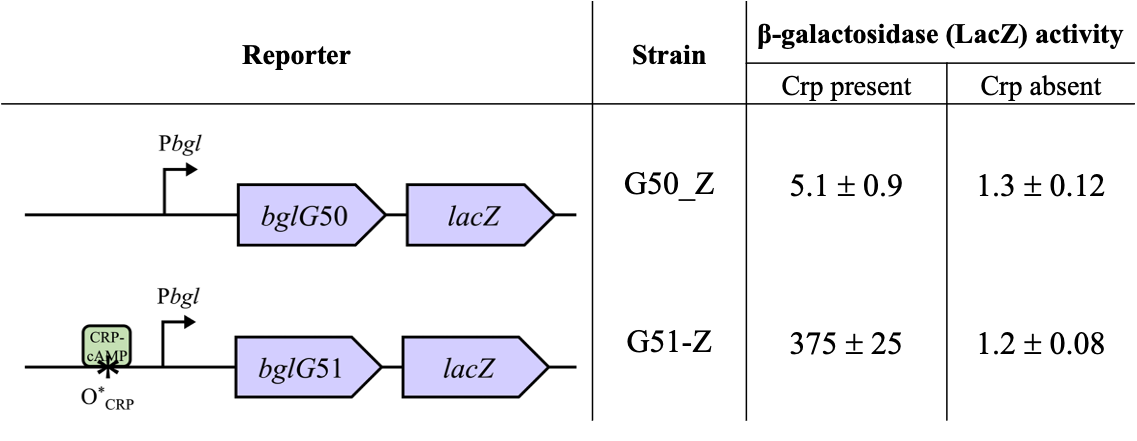


**Figure S3. Effect of Crp binding to the *bgl* operon on *bgl* operon expression.** The β-galactosidase (LacZ) activities of operon reporters G50-Z and G51-Z were measured in the presence or absence of Crp.


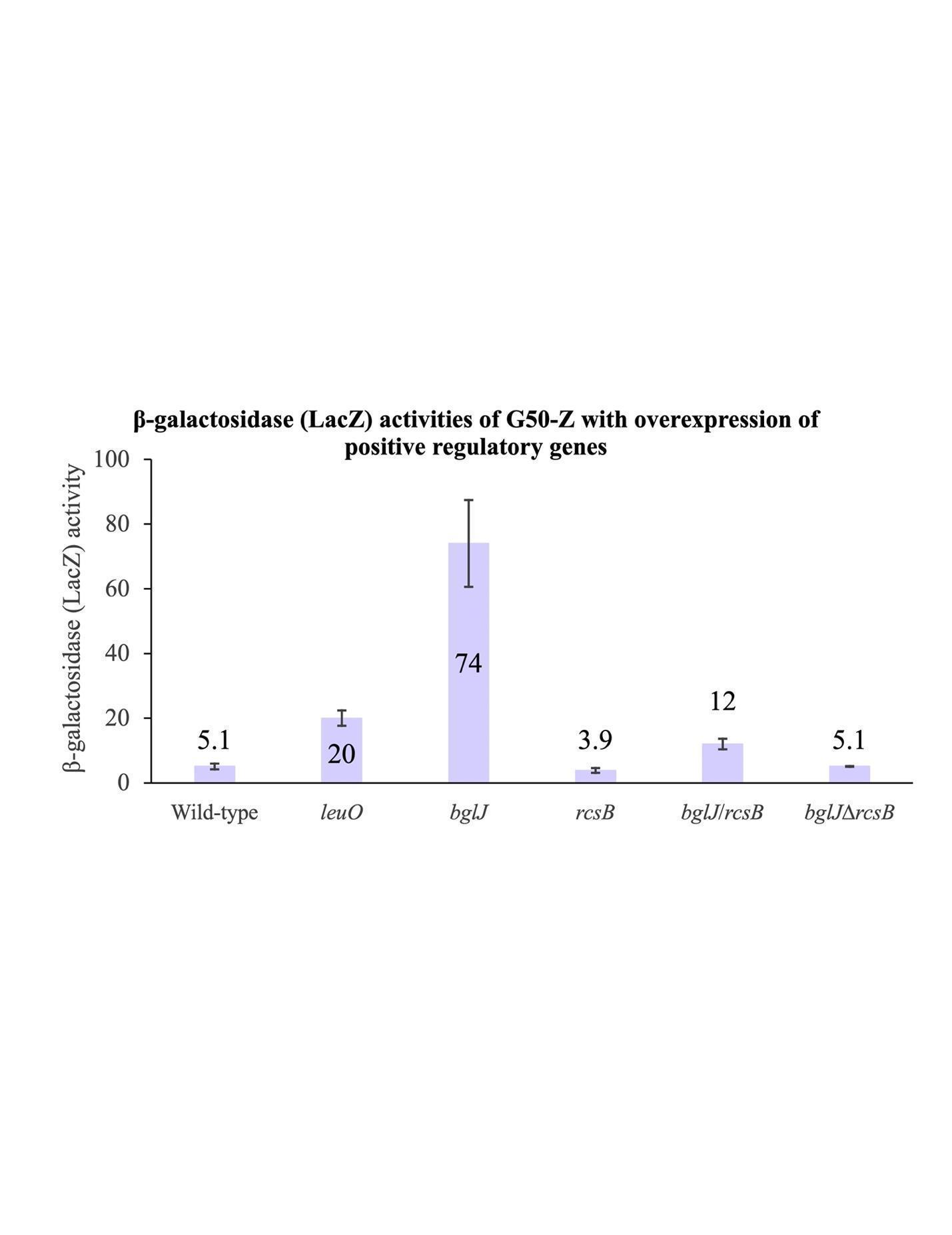


**Figure S4. Effects of overexpression of *leuO, bglJ, rcsB* or both *bglJ*/*rcsB* on *bgl* operon expression in strain G50-Z.** The β-galactosidase (LacZ) activities of reporter G50-Z were determined with overexpression of positive regulators of the *bgl* operon.


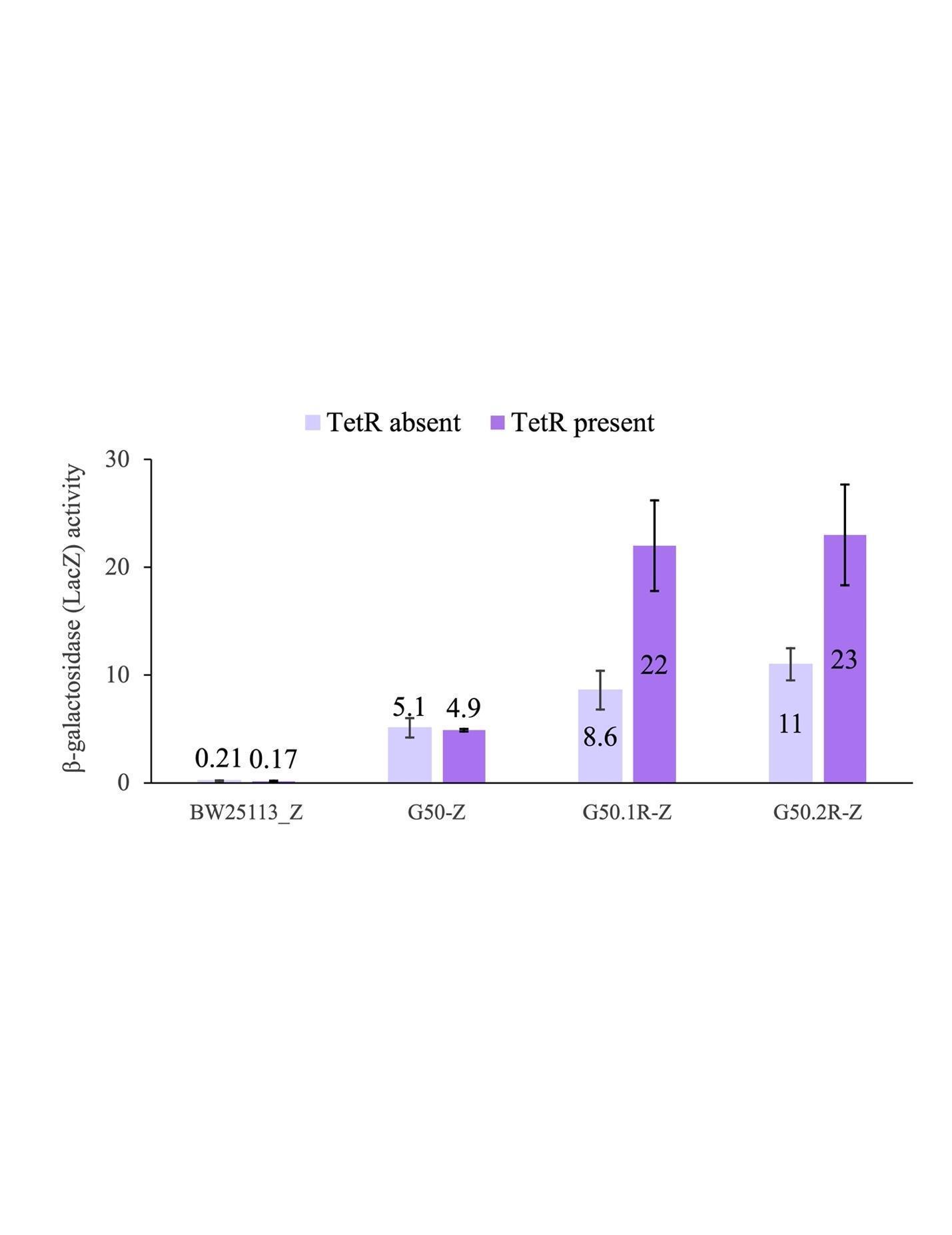


**Figure S5. Effects of replacing the proposed H-NS binding site within *bglG* with one or two TetR binding sites on *bgl* operon expression.** The operon *lacZ* reporter is the same as in strain G50-Z except that the proposed H-NS binding site was replaced by one or two TetR operator sequences.


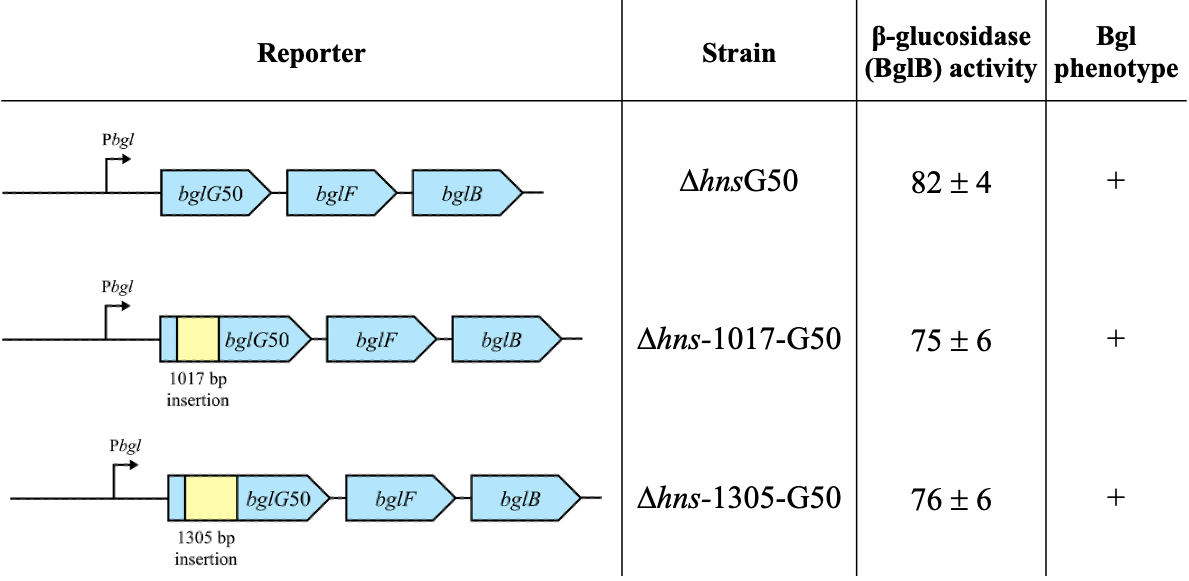


**Figure S6. No terminator is present in the DNA fragments inserted into the *bglG*50 gene.** The left panel shows the operon reporters with or without insertions of the two DNA fragments (1017 bp and 1305 bp) into *bgl*G50 in the background of the *hns* deletion. The right panel shows the operon activities and the Bgl phenotype.

**Supplementary Table 1. Strains and plasmids used in this study**

| **Strains or plasmids** | **Genotype or description** | **Reference or source** |
| --- | --- | --- |
| **Strains** |  |  |
| BW25113 | *E. coli* K12 strain derivative | [67] |
| BW-RI | BW25113 constitutively expressing *tetR* | This study |
| ΔP*bglG* | Deletion of P*bgl*, *bglG* and two terminators flanking *bglG* | This study |
| G50 | Deletion of the two terminators flanking *bglG* | This study |
| RIG50 | G50 constitutively expressing *tetR* | This study |
| ∆*hns* | *hns* deletion in BW25113 | [39] |
| ∆*stpA* | *stpA* deletion in BW25113 | [39] |
| ∆*hns*∆*stpA* | *hns* and *stpA* double deletions in BW25113 | [39] |
| *hns*L30P | Mutation of leucine to proline at 30th codon of *hns* | [39] |
| G50∆O_HNS_-Pbgl | Removal of H-NS binding site at the *bgl* regulatory region in G50 | This study |
| IS5r | IS5 element inserted in reverse orientation at position -207.5 in relation to the *bglG* translational initiation site | This study |
| IS5r∆*hns* | Deletion of *hns* in IS5r | This study |
| IS1r(-236.5) | IS1 element inserted in reverse orientation at position -236.5 in relation to the *bglG* translational initiation site | This study |
| IS1r(-231.5) | IS1 element inserted in reverse orientation at position -231.5 in relation to the *bglG* translational initiation site | This study |
| IS1d(-230.5) | IS1 element inserted in direct orientation at position -230.5 in relation to the *bglG* translational initiation site | This study |
| IS5r(-224.5) | IS5 element inserted in reverse orientation at position -224.5 in relation to the *bglG* translational initiation site | This study |
| IS5d(-207.5) | IS5 element inserted in direct orientation at position -207.5 in relation to the *bglG* translational initiation site | This study |
| IS5r(-207.5) | IS5 element inserted in reverse orientation at position -207.5 in relation to the *bglG* translational initiation site | This study |
| IS5d(-206.5) | IS5 element inserted in direct orientation at position -206.5 in relation to the *bglG* translational initiation site | This study |
| IS2d(-206.5) | IS2 element inserted in direct orientation at position -206.5 in relation to the *bglG* translational initiation site | This study |
| IS2r(-205.5) | IS2 element inserted in reverse orientation at position -205.5 in relation to the *bglG* translational initiation site | This study |
| G51 | G50 with a single mutation at the Crp binding site at the *bgl* regulatory region | This study |
| G50P*tet*-*bglJ* | Overexpression of *bglJ* in G50 | This study |
| G50P*tet*-*bglJ*-*km*^S^ | Overexpression of *bglJ* in G50; sensitive to kanamycin | This study |
| G50P*tet*-*leuO* | Overexpression of *leuO* in G50 | This study |
| G50P*tet*-*rcsB* | Overexpression of *rcsB* in G50 | This study |
| G50P*tet*-*bglJ*/*rcsB* | Overexpression of *bglJ* and *rcsB* in G50 | This study |
| G50P*tet*-*bglJ*Δ*rcsB* | G50 with deletion of *rcsB* and overexpression of *bglJ* | This study |
| ∆*bglG*50 | Deletion of *bglG* in G50 | This study |
| G50.1R | G50 with H-NS binding site within *bglG* replaced by one TetR binding site | This study |
| G50.2R | G50 with H-NS binding site within *bglG* replaced by two TetR binding sites | This study |
| RIG50.1R | G50.1R constitutively expressing *tetR* | This study |
| RIG50.2R | G50.2R constitutively expressing *tetR* | This study |
| G50.P5 | G50 with 5 base pairs inserted within *bglG* | This study |
| G50.P10 | G50 with 10 base pairs inserted within *bglG* | This study |
| 1017-G | 1017 bp insertion of the *cat* gene in the beginning of *bglG* | This study |
| 85-G | Removal of the *cat* gene from *cat*-G50, leaving an 85-bp scar within *bglG* | This study |
| 1017-G50 | G50 with a 1017 bp insertion of the *cat* gene within *bglG* | This study |
| 1305-G50 | G50 with a 1305 bp insertion of the *km* gene within *bglG* | This study |
| BW25113_Z | The *bgl* promoter followed by *bglG* driving *lacZ* gene expression | [51] |
| G50-Z | The *bgl* promoter followed by *bglG* without the two terminators, driving *lacZ* gene expression | This study |
| Ptet-G | The *tet* promoter driving *bglG* at the *intS* locus | [51] |
| Iq-G_Z | The *lacIQ* promoter followed by *bglG* driving *lacZ* gene expression at the *lac* locus | [51] |
| ∆*hns*_Iq-G-Z | Iq-G_Z with deletion of *hns* | This study |
| *hns*L30P_Iq-G-Z | Iq-G_Z with a mutation of leucine to proline at 30th codon of *hns* | This study |
| P*tet*-G_Z | The *tet* promoter followed by *bglG* driving *lacZ* gene expression at the *lac* locus | [51] |
| ∆*hns*_P*tet*-G-Z | P*tet*-G_Z with deletion of *hns* | This study |
| *hns*L30P_P*tet*-G-Z | P*tet*-G_Z with a mutation of leucine to proline at 30th codon of *hns* | This study |
| G51-Z | G50-Z with a single mutation at the Crp binding site at the regulatory region of the *bgl* operon reporter | This study |
| G50-Z-km^S^ | G50-Z; kanamycin sensitive | This study |
| G51-Z-km^S^ | G51-Z; kanamycin sensitive | This study |
| ∆*crp*G50-Z | G50-Z with the removal of *crp* | This study |
| ∆*crp*G51-Z | G51-Z with the removal of *crp* | This study |
| G50-Z-P*tet*-*bglJ* | G50-Z with overexpression of *bglJ* | This study |
| G50-Z-P*tet*-*bglJ*-km^S^ | G50-Z with overexpression of *bglJ*; kanamycin sensitive | This study |
| G50-Z-P*tet*-*leuO* | G50-Z with overexpression of *leuO* | This study |
| G50-Z-P*tet*-*rcsB* | G50-Z with overexpression of *rcsB* | This study |
| G50-Z-P*tet*-*bglJ*/*rcsB* | G50-Z with overexpression of *bglJ* and *rcsB* | This study |
| G50-Z-P*tet*-*bglJ*∆*rcsB* | G50-Z with deletion of *rcsB* and overexpression of *bglJ* | This study |
| G50’-Z | The *bgl* promoter followed by a 3’ region-truncated *bglG*, with the H-NS binding site and the two terminators removed, driving *lacZ* gene expression | This study |
| G50.1R-Z | The *bgl* promoter followed by *bglG* without the two terminators, and H-NS binding site within *bglG* replaced by one TetR binding site, driving *lacZ* gene expression | This study |
| G50.2R-Z | The *bgl* promoter followed by *bglG* without the two terminators, and the H-NS binding site within *bglG* replaced by two TetR binding site, driving *lacZ* gene expression | This study |
| RIG50.1R-Z | G50.1R-Z with the presence of *tetR* | This study |
| RIG50.2R-Z | G50.2R-Z with the presence of *tetR* | This study |
| ∆*hns*G50 | G50 with deletion of *hns* | This study |
| ∆*hns*-1017-G50 | 1017-G50 with deletion of *hns* | This study |
| ∆*hns*-1305-G50 | 1305-G50 with deletion of *hns* | This study |
|  |  |  |
| **Plasmids** |  |  |
| pKES50 | P*bgl* driving a modified *bglG* gene (*bglG*50) without the two terminators | [69] |
| pKDT | The *rrnB* terminator (*rrnBT*) in pKD13 | [70] |
| pKDT_P*bgl*-G50 | P*bgl* driving *bglG*50 in pKDT | This study |
| pKES51 | pKES50 with a point mutation in the Crp-cAMP operator | [69] |
| pKDT_P*bgl*-G51 | pKDT_P*bgl*-G50 with a point mutation in the Crp-cAMP operator | This study |
| pKD4 | Template for FRT-flanked *km*^r^ | [67] |
| pCP20 | For removing *km*^r^ gene; ampicillin and chloramphenicol resistance | [67] |
| pKDT:Ptet | The *rrnBT* followed by P*tet* | [70] |
| pKDT_P*bgl*-G50.5bp | pKDT_P*bgl*-G50 with a 5 bp insertion in *bglG50* | This study |
| pKDT_P*bgl*-G50.10bp | pKDT_P*bgl*-G50 with a 10 bp insertion in *bglG50* | This study |
| pKDT_P*bgl*-G50.1R | pKDT_P*bgl*-G50 with H-NS binding site within *bglG* replaced by one TetR operator | This study |
| pKDT_P*bgl*-G50.2R | pKDT_P*bgl*-G50 with H-NS binding site within *bglG* replaced by two TetR operators | This study |

**Supplementary Table 2. Oligonucleotides used in this study**

| **Name** | **Sequence** | **Use** |
| --- | --- | --- |
| Pbglcat-P1 | taagtctggagtcgctgggccgtcataccatccagatgctgcacgacgtgctgtgtaggctggagctgcttcg | Construction of ∆P*bglG* and G50∆O_HNS_-P*bgl* |
| bglFcat-P2 | caatgttatctgcgcccccgactcctgcgactatttttctggctaactccgtcatatgaatatcctccttagttc | Construction of ∆P*bglG* |
| bglF-ver-R4 | tggttacctatgaccacctgaaactg | Verification for ∆P*bglG* |
| Pbgl-Xh-Fbg | atactcgagcggatggacattgacgaagcggtacg | Construction of pKDT_P*bgl*-G50, G50.1R, G50.2R, G50.P5 and G50.P10 |
| bglG-Bm-Rm | ttaggatccatagctgtttcctgtgaagctatttcagtg | Construction of pKDT_P*bgl*-G50, G50.1R, G50.2R, G50.P5 and G50.P10 |
| Pbgl.G50-P1 | taagtctggagtcgctgggccgtcataccatccagatgctgcacgacgtgctgtgtaggctggagctgcttcg | Construction of G50 and G51, G50.P5 and G50.P10 |
| bglF.G50-P2 | caatgttatctgcgcccccgactcctgcgactatttttctggctaactccgtcatagctgtttcctgtgaagctatttcagtg | Construction of G50, G51, G50.P5 and G50.P10 |
| Pbgl2-P2 | gaaaatagcaatgcgctattgataaaaatatgaccatgctcgcagttattaacatatgaatatcctccttagttc | Construction of G50∆O_HNS_-P*bgl* |
| Pbgl-F2 | tggcgatgagctggataaactgctg | Amplification of *bgl* regulatory region |
| Pbgl-R2 | tcagttcatgactgctcaaggcatac | Amplification of *bgl* regulatory region |
| Ptet-bglJ-P1 | tgatatgaaagtgaatgctaaggataatttattcgcttaatctattaatttgtgtaggctggagctgcttc | Construction of P*tet*-*bglJ* |
| Ptet-bglJ-P2 | cataacaggtattttactgataacaattccatttttgcagcatcctggcaacatggtacctttctcctctttaatgaattc | Construction of P*tet*-*bglJ* |
| bglJ-ver-R | aacagaacgtggatcttcactttcac | Verification of P*tet*-*bglJ* |
| Ptet-leuO-P1 | ttatggattattatgctgtggtaaatgactcattccacggcaatggattctgtgtaggctggagctgcttc | Construction of P*tet*-*leuO* |
| Ptet-leuO-P2 | ggtttgcttaactccgccgtctctggatgatctgtttgtacctctggcatggtacctttctcctctttaatgaattc | Construction of P*tet*-*leuO* |
| leuO-ver-R | tgttttgctcctgcatcacggcatcg | Verification of P*tet*-*leuO* |
| Ptet-rcsB-P1 | gaaaaatacatcagcgacattgacagttatgtcaagagcttgctgtagcaagtgtgtaggctggagctgcttc | Construction of P*tet*-*rcsB* |
| Ptet-rcsB-P2 | gaacaagactatcggatggtcatcggcaataattacgttcatattgttcatggtacctttctcctctttaatgaattc | Construction of P*tet*-*rcsB* |
| rcsB-ver-R | tacttatcgccaggcatggagagatcg | Verification of P*tet*-*rcsB* |
| Ptet-Xh-F | atactcgagactctatcattgatagag | Cloning of P*tet* |
| G50-P1 | cctggatgttcgttataaaaaccattaataaatgactggattgttactgctgtgtaggctggagctgcttc | Construction of Δ*bglG*50 |
| G50-P2 | gactatttttctggctaactccgtcataacttgccctctaccgctttgcggattccggggatccgtcgacctg | Construction of Δ*bglG*50 |
| Ohns-F1 | gttaatgcgcgtccctatcagtgatagagaatttcagttcagccttaattaccaggaag | Construction of G50.1R |
| Ohns-R1 | ctctatcactgatagggacgcgcattaactgcgtgacacctgcaac | Construction of G50.1R |
| bglG.2R-F | gaatttcagttcagccttaattaccaggaagaaagcttg | Construction of G50.2R |
| bglG.2R-R | gactgcaacatcctccatatttccgctc | Construction of G50.2R |
| 2R-F | gtgcccaaatgagcggaaatatggaggatgttgcagtc | Construction of G50.2R |
| G50-cat-P1 | cagtcatgaactgaacgggcgattaagcgaactcttaagtcatattcctcttgtgtaggctggagctgcttcg | Construction of 1017-G, 1017-G50, G50.P5 and G50.P10 |
| G50-cat-P2 | caagcgctcctgcgctaaagagataatacgatcacaggttgccatcacctcacatatgaatatcctccttagttc | Construction of 1017-G and 1017-G50 |
| G50-pha5-F | ctttcaaaaacgcatagagctggcgaaagaattaactcaagtgg | Construction of G50.P5 |
| G50-pha5-R | gagttaattctttcgccagctctatgcgtttttgaaagccaattcc | Construction of G50.P5 |
| G50-pha10-F | gctttcaaaaacgcatagaatagagctggcgaaagaattaactcaagtgg | Construction of G50.P10 |
| G50-pha10-R | ctttcgccagctctattctatgcgtttttgaaagccaattccgc | Construction of G50.P10 |
| Pu1n-P1n | gcatttacgttgacaccatcgaatggcgcaaaacctttcgcggtatgtgtaggctggagctgcttc | Construction of all *lacZ* reporters |
| Pbgl.dT-GZ-P2 | cgacggccagtgaatccgtaatcatggtcatagctgtttcctgtgtgaaatttcagtgttctttgcgcacgcgctctat | Construction of G50-Z and G51-Z |
| G50tn-Z-P2 | gttgtaaaacgacggccagtgaatccgtaatcatggtcatagctgtttcctgtgaagcttagcccacttcatctttcggtaactg | Construction of G50T-Z |
